# Malic enzyme 1 contributes to tumorigenesis and lenvatinib resistance in hepatocellular carcinoma via FSP1-dependent ferroptosis evasion

**DOI:** 10.1038/s41419-026-08572-w

**Published:** 2026-03-25

**Authors:** Danyi Wu, Huanhuan Xu, Yi Guo, Lulu Lu, Dan Han, Luyi Chen, Ruoxuan Lei, Min Li, Wei Wu, Wen-Zhuo He, Yingying Yu, Xuexian Fang

**Affiliations:** 1 School of Public Health and Nursing, Zhejiang Key Laboratory of Medical Epigenetics, Hangzhou Normal University, Hangzhou, China; 2 Department of Nutrition and Food Safety, Zhejiang Provincial Center for Disease Control and Prevention, Hangzhou, China; 3 Department of General Practice, Sir Run Run Shaw Hospital, Zhejiang University School of Medicine, Hangzhou, China; 4 Sun Yat-sen University Cancer Center, Guangzhou, China

**Keywords:** Cancer metabolism, Cell death

## Abstract

Hepatocellular carcinoma (HCC) is the most prevalent hepatic malignancy worldwide, accounting for approximately 90% of all primary liver cancer cases. However, the mechanisms involving in liver tumorigenesis and drug resistance remain unclear, largely restricting the clinical management of HCC. Here, we first evaluated the clinical significance of malic enzyme 1 (ME1) in HCC patients and revealed that ME1 was significantly upregulated in tumor tissues and positively correlated with poor prognosis. Gain- and loss-of-function experiments suggested that ME1 promoted HCC cell viability in vitro. Consistently, hepatocyte-specific *Me1* knockout (*Me1*^*HKO*^) mice treated with diethylnitrosamine (DEN) showed reduced tumor burden as compared to *Me1*^*Flox*^ mice. In addition, *ME1* overexpression conferred resistance to the first-line therapeutic drug lenvatinib, while knockout of *ME1* restored drug sensitivity in lenvatinib-resistant HCC cells. Mechanistically, we showed that ME1 could regulate ferroptosis of HCC cells through its function on NADPH production. We further identified ferroptosis suppressor protein 1 (FSP1) as a key downstream effector, which utilized ubiquinol (CoQH_2_) as a lipophilic radical-trapping antioxidant to block the accumulation of lipid peroxides to pro-ferroptotic levels. In summary, our findings demonstrated that ME1 promotes HCC progression by activating the NADPH-FSP1-CoQH_2_ axis and thereby inhibiting ferroptosis, suggesting a promising therapeutic strategy for HCC treatment.

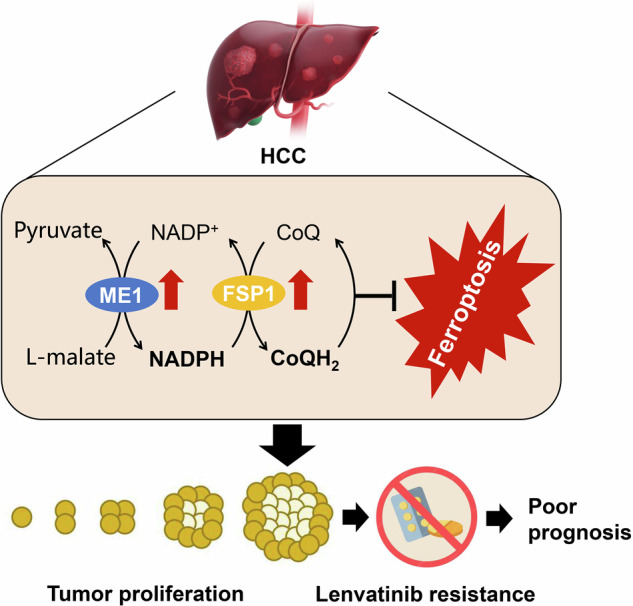

## Introduction

Liver cancer constitutes a global health concern with increasing incidence and ranks among the top three cancers for mortality worldwide [1]. Hepatocellular carcinoma (HCC) represents the most prevalent type of primary liver cancer [2]. Although infection with hepatitis B or hepatitis C virus is still the dominant risk factor for tumor development, metabolic dysfunction-associated steatohepatitis and alcohol-associated liver disease are becoming the fastest-rising causes of HCC [3]. Early-stage HCC could be well treated with curative surgery, involving either hepatic resection or liver transplantation. Unfortunately, the majority of HCC patients are diagnosed at intermediate or advanced stage, missing the optimal period for curative treatment and leading to a worse prognosis [4]. Therefore, it is urgent to understand the molecular mechanisms regulating the initiation and progression of HCC and uncover appropriate therapeutic targets for clinical translation.

Lenvatinib (E7080/MK-7902), a multi-targeted tyrosine kinase inhibitor, is the second approved first-line drug for the therapy of unresectable advanced HCC after sorafenib [5]. Lenvatinib is superior to sorafenib with longer overall as well as progression-free survival and higher objective response rate in HCC patients, with similar safety and tolerability profiles [6, 7]. However, over 60% of patients will develop resistance to lenvatinib during treatment and suffer subsequent HCC progression, severely limiting its efficacy and clinical application [8]. Thus, investigations on the underlying mechanisms of lenvatinib resistance and biomarkers predicting lenvatinib response are urgently needed.

Malic enzyme 1 (ME1) is a cytosolic NADP^+^-dependent enzyme that catalyzes the formation of pyruvate from malate and provides reducing power in the form of nicotinamide adenine dinucleotide phosphate (NADPH)[9]. Its activation has been associated with accelerated tumor growth in gastric, colorectal, and breast cancers [10–12]. Previously, we demonstrated that ME1 is a key regulator of hepatic ferroptosis, an iron-dependent form of regulated cell death [13]. Ferroptosis has been associated with poor prognosis and resistance to targeted therapy in HCC [14, 15]. But whether ME1-mediated ferroptotic cell death associates with HCC tumorigenesis and lenvatinib resistance remains largely unknown.

In this study, we report a tumor-promoting role of ME1 in HCC by suppressing ferroptosis through NADPH-dependent activation of ferroptosis suppressor protein 1 (FSP1). In addition, high ME1 expression also contributes to the poor therapeutic efficacy of lenvatinib. Loss of hepatic Me1 in mice restricts FSP1 activity, thereby promoting ferroptosis to repress tumor progression. Our findings demonstrate that HCC is dependent on ME1-mediated ferroptosis evasion, and that inhibiting ME1/FSP1 activity represents a promising approach to suppress HCC development and overcome drug resistance.

## Materials and Methods

### Mice

All experiments involving animals were performed in accordance with the National Institutes of Health’s Guide for the Care and Use of Laboratory Animals and were approved by the Animal Care and Use Committee of Hangzhou Normal University (NO. HSD-20240830-01). BALB/c nude mice were purchased from GemPharmatech (Nanjing). For subcutaneous tumor xenograft models, 5 × 10^6^ HepG2 cells (with or without *ME1* overexpression) were subcutaneously injected into the right flank of nude mice. Xenograft volume was measured by caliper and recorded twice a week.

Hepatocyte-specific *Me1* gene deletion (*Me1*^*HKO*^) mice were generated as previously described [13]. For induction of HCC, male *Me1*^*HKO*^ and littermate control mice were administered a single dose of DEN (40 mg/kg body weight) at postnatal day 14. These mice were sacrificed after 8 months.

### Human liver samples

HCC tissues (*n* = 79) and paired adjacent tissues (*n* = 16) were collected from patients with HCC at the Sir Run Run Shaw Hospital, Zhejiang University School of Medicine (Hangzhou, China). The collection and use of these clinical samples was conducted in accordance with Declaration of Helsinki and had been approval by the Ethics Committee of Sir Run Run Shaw Hospital (No. 2024-2672-01) with informed consent from patients.

### Cell lines and cell culture

Human HCC cell lines (HepG2, Huh7 and Huh1) were purchased from the Cell Bank of the Chinese Academy of Science (Shanghai). All cell lines were confirmed via STR profiling. Cells were cultured in DMEM (Sangon Biotech) supplemented with 10% fetal bovine serum (FBS; Sangon Biotech), 100 IU/mL penicillin, and 100 IU/mL streptomycin. All cells were grown at 37 °C in a humidified incubator containing 5% CO_2_. All chemicals are listed in Supplementary Table 1.

### Overexpression and knockdown of ME1

ME1 stable overexpression was achieved by using lentiviral technology (Ubigene, Guangzhou). Short hairpin RNA (shRNA) targeting ME1 (shME1) and negative control shRNA (shCtrl) were synthesized by Quanyang (Shanghai). Cells were transfected with 0.8 μg shME1 or shCtrl using Lipofectamine 3000 (Thermo Fisher) transfection reagent in accordance with the manufacturer’s protocol. The sequences are listed in Supplementary Table 2.

### Establishment of lenvatinib-resistant HCC cells

For achieving lenvatinib-resistant HCC cells, Huh7 cells were exposed lenvatinib (S1164, Selleck) at an initial dose just below the half-maximal inhibitory concentration (IC50), and gradually increased by 1 μM per week for about 6–8 months. After establishment, these resistant HCC cells were continuously cultured in the presence of 10 μM lenvatinib.

### Cell viability

Cell viability was determined by cell counting kit-8 (CCK-8) assay (Meilunbio). Cells (2 × 10^3^ cells/well) were plated in 96 well plates with 100 μL of culture medium and cultured for the indicated time. With supernatant removed, CCK-8 reagent was added to each well with 10 μL land incubated for 1 h at 37 °C. The absorbance at 450 nm was detected using a microplate reader (Molecular Devices).

### Dead cell staining

Cell death was also assessed by labeling with propidium iodine (PI; Lablead) and imaged by a microscopy system from Olympus.

### Colony formation assay

Cells (1×10^3^/well) were seeded into 6 well plates and cultured for 2 weeks to allow colony formation. During this period, the fluid was changed with fresh DMEM medium every 3 days. Colonies were fixed with 4% polyoxymethylene and then stained with crystal violet solution.

### Migration assay

Cells (5 × 10^4^/well) were seeded in 6-well plates at an appropriate density to ensure that they reach 80–90% confluence within 24 h. A wound was created in each well by scraping the cells with a sterile 200 μL pipette tip. Consistent pressure was applied to ensure a uniform wound width. Images were taken at appropriate time intervals (e.g., 0, 6, 12, 24, 48 h) depending on the cell migration rate.

### Quantitative real-time PCR

Total RNA was extracted from cells or tissues using TRIzol (Pufei) and complementary DNA was synthesized with Strand cDNA Synthesis SuperMix (Yeasen). Quantitative real-time PCR was performed using SYBR Green Master Mix (Yeasen). All samples were normalized to the expression of GAPDH. Primer sequences are shown in Supplementary Table 2.

### Immunoblotting

Total protein was extracted from the tissue or cells by homogenization in RIPA buffer with protease inhibitor cocktail (Meilunbio). Extracted proteins were separated by electrophoresis through SDS-PAGE and transferred to PVDF membranes (Millipore). After blocking with 5% nonfat milk, the membranes were incubated overnight at 4 °C with primary antibodies and then incubated with HRP-conjugated secondary antibodies (Proteintech) for 2 h. The blots were detected with an ECL detection reagent (Bio-Rad). Antibodies are listed in Supplementary Table 3.

### Histological analysis

Serial sections of tissues were performed after overnight fixation in 4% paraformaldehyde and embedding in paraffin. Upon staining with hematoxylin and eosin (H&E) or Sirius Red, the sections were examined under a light microscope (Olympus).

### Immunohistochemistry (IHC)

Deparaffinized serial sections were blocked with PBS containing 5% goat serum and 1% BSA, and then incubated overnight at 4 °C with anti-4-hydroxynonenal (4-HNE) antibody (ab46545; Abcam), anti-ME1 antibody (ab97445), or anti-FSP1 antibody (a22278, ABclonal). This was followed by a 1 h incubation with secondary antibody (Proteintech) at room temperature.

### Transmission electron microscopy (TEM)

Cells were fixed in 2.5% glutaraldehyde at 4 °C. After fixation, the samples were dehydrated using ethanol gradients, followed by acetone incubation and embedding in ethoxyline resin. Ultrathin sections were cut and observed by a Hitachi TEM at 120 kV.

### Measurement of liver function

Serum levels of ALT, AST, and LDH were measured by using an automatic biochemical analyzer (Sysmex).

### Measurement of alpha-fetoprotein (AFP)

Serum AFP levels were measured by a commercial kit (JHN80018, JinHengNuo).

### Measurement of MDA

The intracellular MDA levels were measured by a commercial kit (A003, Jiancheng Bioengineering).

### Measurement of NADPH/NADP^+^

The intracellular levels of NADPH and NADP^+^ were measured by a commercial kit (S0179, Beyotime).

### Measurement of CoQH_2_/CoQ

The intracellular levels of CoQH_2_ and CoQ were measured by ultra-high performance liquid chromatography-tandem mass spectrometry (UHPLC-MS/MS). The platform utilized in the project was an Agilent 1290 Infinity II UHPLC coupled to an Agilent 6470 A Triple Quadrupole MS.

### Measurement of oxylipins

Oxylipins were analyzed as previously described [16].

### Statistics

Statistical analyses were performed using GraphPad Prism software version 9.0 (GraphPad Software Inc, California). Sample sizes were determined via power analysis (*α* = 0.05, 80% power) using data from prior studies and pilot experiments. After genotyping, mice were randomly assigned to treatment groups. To minimize observer bias, all experimental procedures were conducted under blinded conditions. All summary data were presented as the mean ± SEM. Groups were compared using the two-tailed Student’s *t*-test. The difference was considered statistically significant with a *P* value of less than 0.05.

## Results

### ME1 is up-regulated in HCC and predicts poor prognosis

**Fig. 1:**
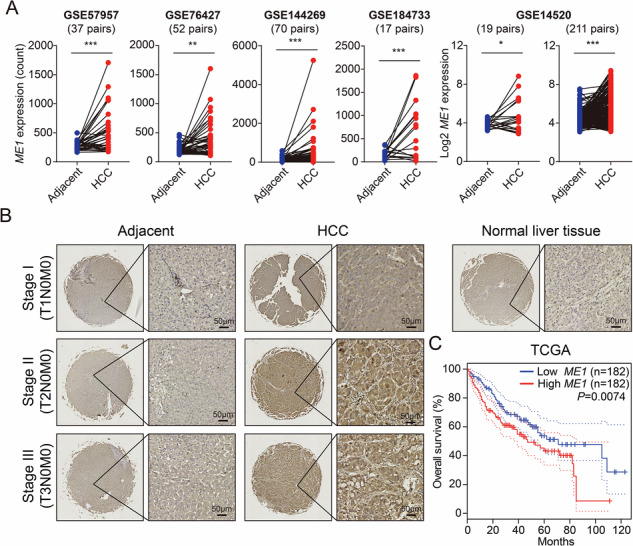
ME1 is upregulated and associated with poor prognosis in HCC. **A** Based on the GEO database, five datasets (GSE57957, GSE76427, GSE144269, GSE184733, GSE14520) were analyzed to compare *ME1* mRNA expression between HCC tumor tissues and adjacent non-tumor tissues. **B** Representative immunohistochemistry images of ME1-stained sections from HCC tissues (*n* = 79) and paired adjacent tissues (*n* = 16). **C** Kaplan-Meier overall survival curves for liver cancer patients from the TCGA database (*n* = 182), stratified by the median expression level of ME1. Data were calculated by unpaired two-tailed Student’s *t*-test (A) or Kaplan-Meier analysis with the log-rank test (**C**); *P < 0.05, **P < 0.01, and ***P < 0.001.

To explore the expression of ME1 in HCC, we first analyzed multiple sets of data from GEO database and identified that *ME1* mRNA expression was elevated in HCC tissues compared with adjacent non-tumor tissues of patients (Fig. 1A). ME1 protein expression profiling was further confirmed by immunohistochemistry in HCC clinical specimens. As shown in Fig. 1B, HCC tumors of stage II and III exhibited a significant increase in the number of hepatocytes overexpressing ME1. To determine the prognostic value of *ME1* genes, we performed Kaplan-Meier analysis using TCGA dataset and found that high expression of ME1 was associated with poor overall survival (log rank *P* < 0.01) in HCC patients (Fig. 1C). Collectively, these results indicate that ME1 may play a tumor-promoting role in the development and treatment of human HCC.

### ME1 enhances the viability of HCC cells in vitro and in vivo

**Fig. 2:**
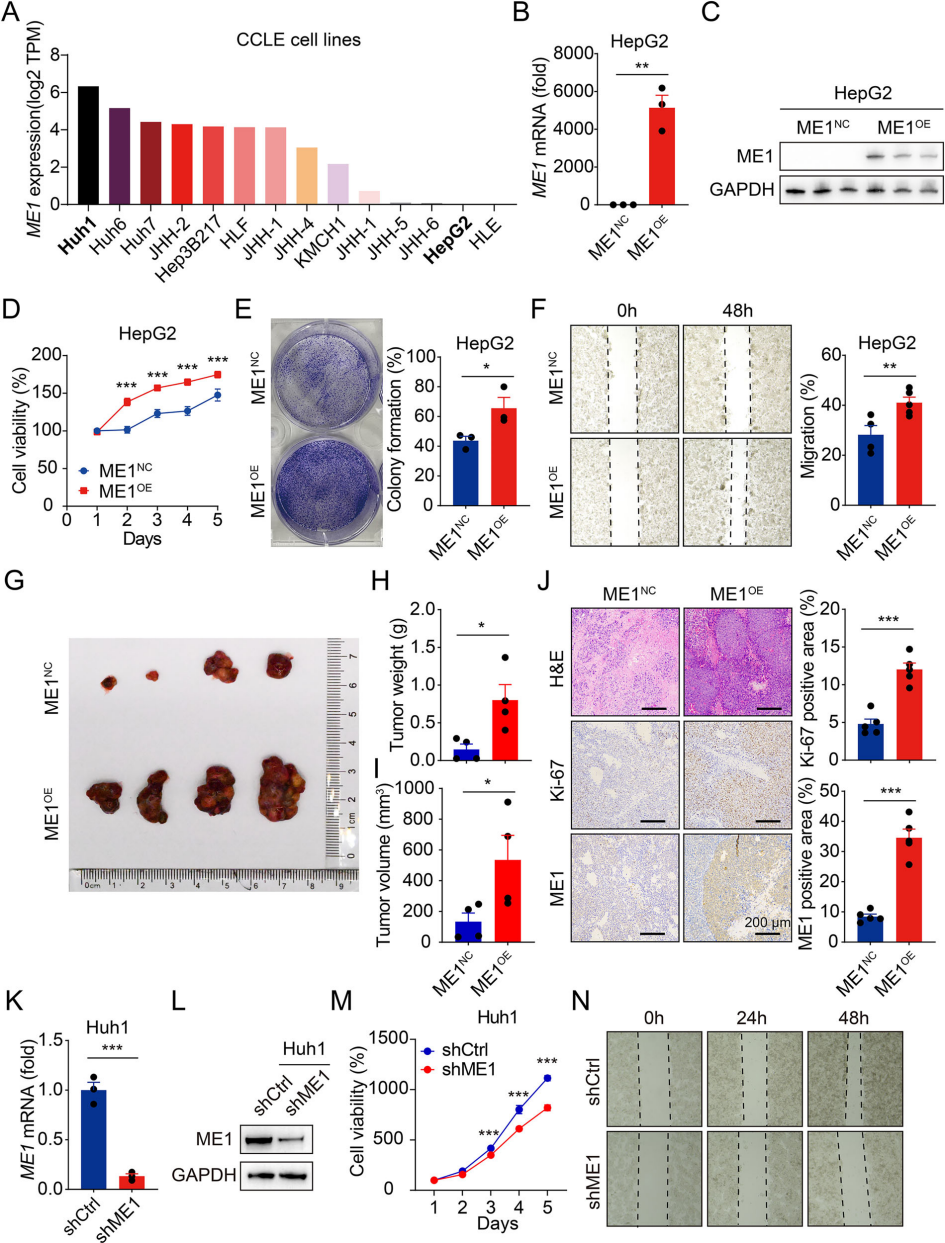
ME1 promotes the viability of HCC cells. **A** ME1 expression in a panel of HCC cells based on the CCLE database. **B**, **C** The stable overexpression of ME1 in HepG2 cells was confirmed by qPCR (**B**) and Western blotting (**C**). Cells transfected with an empty vector (ME1^NC^) served as the control group, while those overexpressing ME1 constituted the ME1^OE^ group. **D** Cell viability was analyzed in HepG2 cells stably transfected with control or ME1 expression plasmids. **E** Colony formation of HepG2 cells stably transfected with control or ME1 expression plasmids was assessed and quantified after 2 weeks. **F** Wound healing assay and quantitative analysis of HepG2 cells stably transfected with control or ME1 expression plasmids at 0 and 48 h. **G** Representative images of xenograft liver tumors from nude mice subcutaneously injected with HepG2 cells stably transfected with control or ME1 plasmids (*n* = 4 per group; tumors harvested at 3 weeks). **H**,**I** Quantitative analysis of tumor weights (**H**) and volumes (**I**) in the xenograft model. **J** Representative images and quantitative analyses of H&E, Ki-67, and ME1 staining in xenograft tumor tissues. **K**, **L** The knockdown efficiency of ME1 in Huh1 cells transfected with shME1 or control shRNA was measured by qPCR (**K**) and western blotting (**L**). **M** Cell viability was analyzed in Huh1 cells stably expressing control or ME1-specific shRNA. **N** Wound healing assay of Huh1 cells transfected with control or ME1 shRNA at 0, 24, and 48 h. Data were calculated by unpaired two-tailed Student’s *t*-test; *P < 0.05, **P < 0.01, and ***P < 0.001.

Next, we examined the expression levels of ME1 in HCC cell lines of the CCLE database and identified high ME1 expression in Huh1 and Huh6 cells and low expression in HepG2 and HLE cells (Fig. 2A). To investigate the effect of ME1 on the function of HCC cells, we first generated HepG2 cell line stably overexpressing ME1 (Fig. 2B, C). Cell viability and colony formation assays showed that overexpression of ME1 significantly promoted cell viability and enhanced clonogenic capacity (Fig. 2D, E). In addition, the migration ability in ME1-overexpressed HCC cells was also activated, as showed by wound healing assay (Fig. 2F). To further explore the role of ME1 on tumor growth in vivo, we established a subcutaneous xenograft tumor model in nude mice. Consistent with the in vitro observations, overexpression of ME1 remarkably accelerated HepG2 xenograft tumor growth, with increased cancer cell proliferation (with the marker Ki-67) (Fig. 2G–I).

Furthermore, we constructed a shRNA plasmid targeting ME1 and transfected it into Huh1 cell line, which has relatively high basal ME1 expression (Fig. 2J, K). Knockdown of ME1 significantly suppressed cell migration of Huh1 cells (Fig. 2L, M and Supplementary Fig. 1). Together, these data suggest that ME1 facilitates HCC development.

### *Me1* deletion represses tumorigenesis in an HCC mouse model

**Fig. 3:**
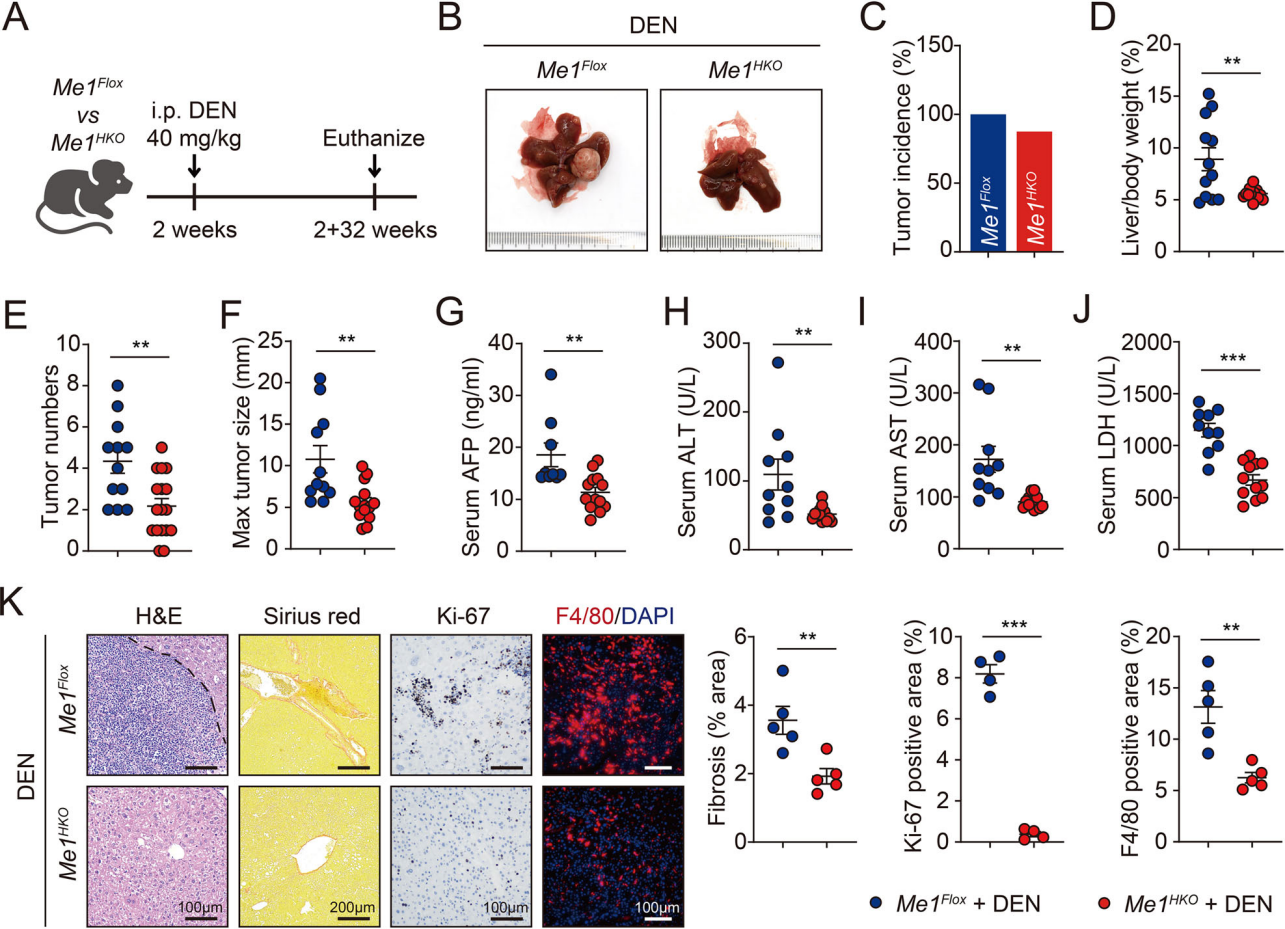
Hepatocyte-specific *Me1* deletion restrains tumorigenesis in DEN-induced HCC mouse model. **A** Schematic of the DEN-induced HCC mouse model. *Me1*^*HKO*^ and control (*Me1*^*Flox*^) mice were injected intraperitoneally with a single dose of DEN (40 mg/kg) at two weeks of age, then mice were maintained under SPF conditions for 32 weeks prior to euthanasia and tissue harvest. **B** Representative images of *Me1*^*Flox*^ and *Me1*^*HKO*^ livers tumors at 32th week after DEN injection. **C–F** The tumor incidence (**C**), the liver-to-body weight ratio (**D**), the number of tumors per liver (**E**), and the maximum tumor diameter (**F**) were assessed in *Me1*^*Flox*^ and *Me1*^*HKO*^ mice. **G–J** The serum levels of alpha-fetoprotein (AFP, G), alanine aminotransferase (ALT, H), aspartate aminotransferase (AST, I), and lactate dehydrogenase (LDH, J) were measured in *Me1*^*Flox*^ and *Me1*^*HKO*^ mice. **K** Representative images and corresponding quantitative analyses of H&E, Sirius Red, Ki-67, and F4/80 staining in liver tissues from *Me1*^*Flox*^ and *Me1*^*HKO*^ mice. Data were calculated by unpaired two-tailed Student’s *t*-test; *P < 0.05, **P < 0.01, and ***P < 0.001.

We next produced hepatocyte-specific *Me1* knockout (*Me1*^*HKO*^) mice (Supplementary Fig. 2A) and investigated the tumor-promoting effect of ME1 on chemical carcinogen diethylnitrosamine (DEN)-induced HCC development in vivo (Fig. 3A). Although tumor incidence remained substantial, *Me1*^*HKO*^ mice exhibited significantly reduced tumor burden compared to littermate control (*Me1*^*Flox*^) mice, as indicated by restored liver weight, decreased tumor numbers, and smaller tumor size (Fig. 3B–F and Supplementary Fig. 2B). In addition, serum levels of alpha-fetoprotein (AFP), a well-established HCC biomarker, were significantly suppressed in *Me1*^*HKO*^ mice (Fig. 3G). DEN treatment also led to less liver injury, measured by the release of alanine aminotransferase (ALT), aspartate aminotransferase (AST), and lactate dehydrogenase (LDH) to the circulation, in *Me1*^*HKO*^ mice than in controls (Fig. 3H–J). Our histological observation also showed that *Me1*^*HKO*^ mice were resistant to DEN-induced hepatic fibrosis, cancer cell proliferation, and inflammatory infiltration (Fig. 3K and Supplementary Fig. 2C). In short, all these results clearly demonstrate that loss of hepatic *Me1* inhibits HCC development in mice.

### ME1 protects HCC cells against ferroptotic cell death

**Fig. 4:**
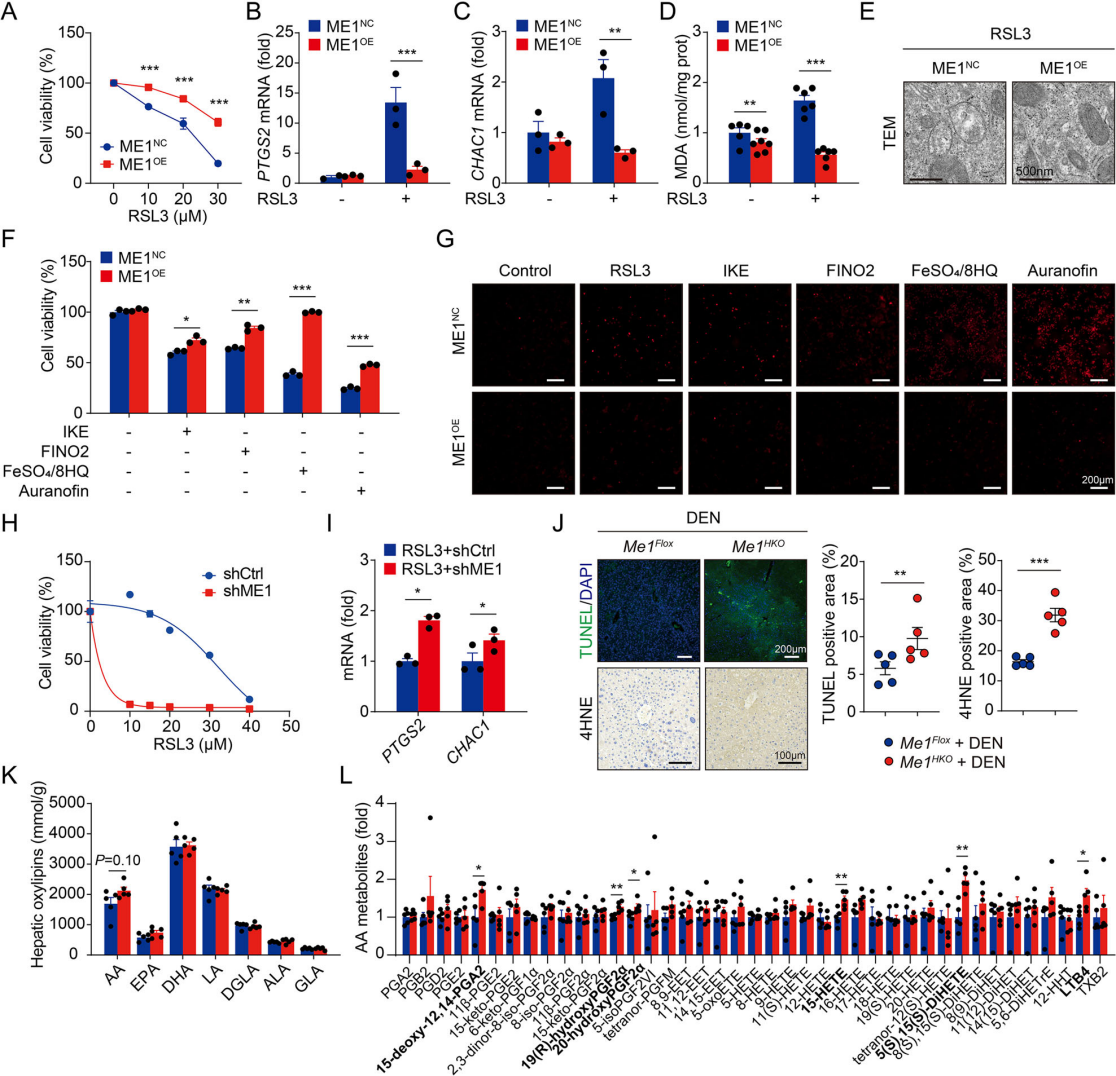
ME1 mediates ferroptosis sensitivity in HCC. **A** Cell viability was measured after 24 h of RSL3 treatment to compare HepG2 cells transfected with a control vector versus an ME1-overexpressing plasmid. **B**–**D** *PTGS2* mRNA (**B**) *CHAC1* mRNA (**C**) and lipid peroxidation byproduct malondialdehyde (MDA) content (**D**) were tested in cells transfected with a control vector versus an ME1-overexpressing plasmid, treated with or without RSL3 (20 μM) for 24 h. **E** The ultrastructural changes in mitochondria, visualized by electron microscopy, are shown in HepG2 cells treated with RSL3 (20 μM) for 24 h after transfection with either a control or an ME1-overexpressing vector. **F**, **G** Cell viability (**F**) and PI staining (**G**) were assessed in HepG2 cells transfected with a control or an ME1 vector, following 24 h treatment with various ferroptosis inducers. The inducers and concentrations used were: IKE (40 μM), FINO₂ (20 μM), FeSO₄/8HQ (2 μM), and Auranofin (8 μM). **H** The viability of control or ME1 shRNA-infected Huh1 cells treated with RSL3 (0-50 µM) for 24 h was assessed. **I** *PTGS2* and *CHAC1* mRNA expression was analyzed and compared in control or ME1 shRNA-infected Huh1 cells after 24 h treatment with RSL3 (20 μM). **J** Representative images and quantitative analyses of TUNEL and 4HNE staining in livers from DEN-induced *Me1*^*Flox*^ and *Me1*^*HKO*^ mice. **K**, **L** A summary of hepatic oxylipin (**K**) and arachidonic acid metabolite (**L**) profiles in DEN-induced *Me1*^*Flox*^ and *Me1*^*HKO*^ mice. Data were calculated by unpaired two-tailed Student’s *t*-test; *P < 0.05, **P < 0.01, and ***P < 0.001.

Previously, we identified ME1 as a potent suppressor of ferroptosis in the liver [13]. Therefore, we hypothesized that resistance to ferroptosis may be the key mechanism driving the tumor-promoting effect of ME1. To address this possibility, ME1-overexpressing HepG2 cells were treated with RSL3, a specific ferroptosis inducer. ME1 overexpression resulted in significant resistance to RSL3-induced ferroptotic cell death (Fig. 4A and Supplementary Fig. 3A). Ferroptosis is driven by lipid peroxidation and activates expression of a series of marker genes like *PTGS2* and *CHAC1* [17, 18]. Consistently, ME1 overexpression significantly restored the marker gene expression and the accumulation of malondialdehyde (MDA), a final product and marker of lipid peroxidation, to a level similar to that in wild-type HCC cells (Fig. 4B–D). Abnormal mitochondrial structure is another important hallmark of ferroptosis. Transmission electron microscopy (TEM) indicated severe mitochondrial distortion after RSL3 treatment, which was markedly attenuated by overexpression of ME1 (Fig. 4E). In addition, we further verified the pro-ferroptotic role of ME1 in HCC cells by parallel treatment of multiple known ferroptosis inducers (Fig. 4F, G and Supplementary Fig. 3B-E).

On the other hand, ferroptosis was readily induced in the HCC cells expressing shME1 (Fig. 4H, I). Consistently, an increased number of terminal deoxynucleotidyl transferase dUTP nick end labeling (TUNEL; a general indicator of cell death) positive foci was found in *Me1*^*HKO*^ livers, with intense 4-hydroxynonenal (4-HNE; a lipid peroxidation product) deposits (Fig. 4J). *Me1*^*HKO*^ mice also showed higher expression of ferroptosis-associated genes in the liver (Supplementary Fig. 2D, E). In addition, we analyzed hepatic oxidized lipids (oxylipins) using UHPLC-MS/MS. And elevated levels of 15-hydroxyeicosatetraenoic acid (15-HETE), an oxidized arachidonic acid (AA) metabolite and reliable marker of ferroptosis, were detected in *Me1*^*HKO*^ livers (Fig. 4K, L). Together, these data suggest that ME1 activation enhances HCC cell survival primarily by conferring resistance to ferroptosis.

### ME1 regulates lenvatinib resistance in HCC by suppressing ferroptosis

**Fig. 5:**
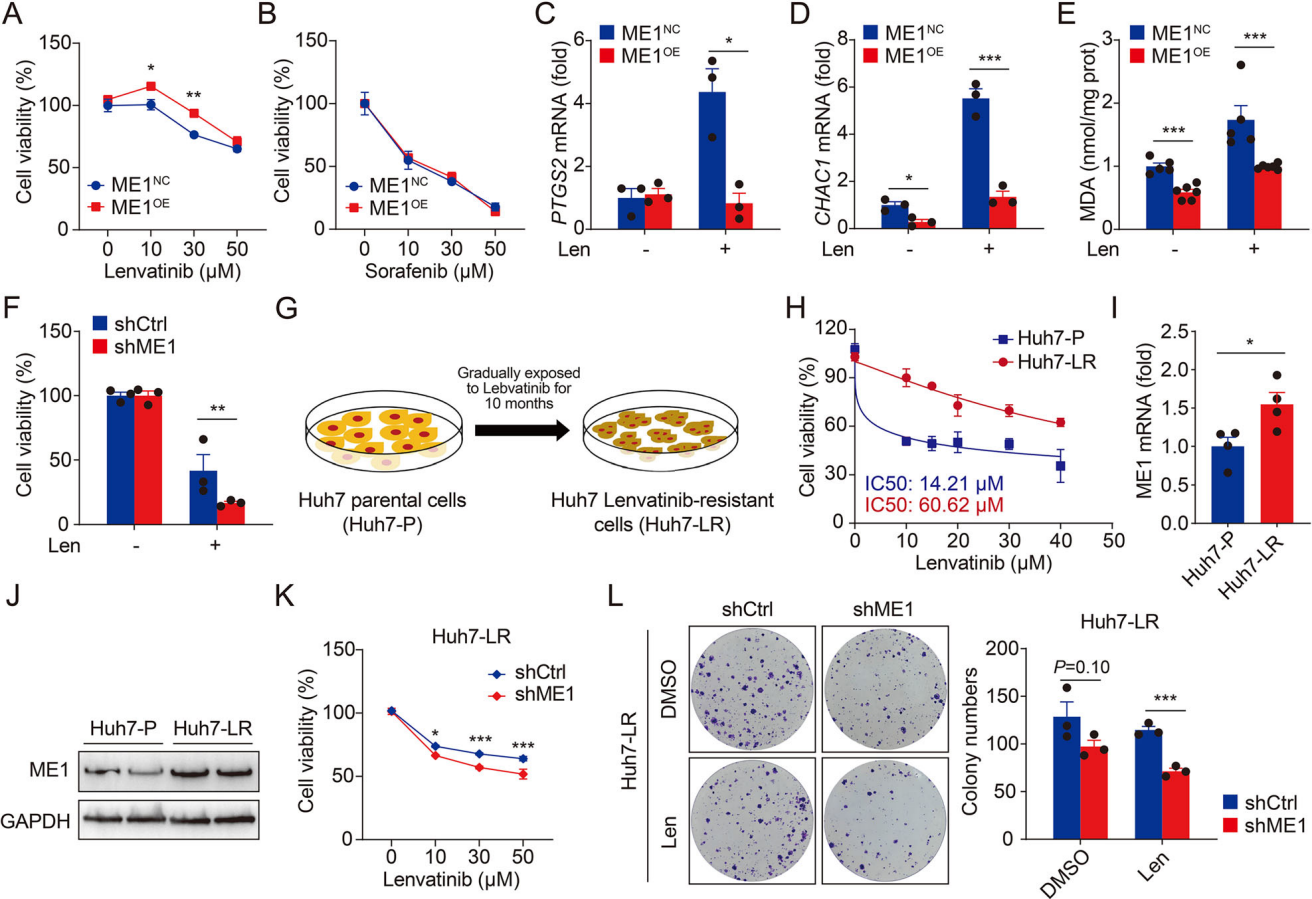
ME1 promotes lenvatinib resistance via ferroptosis regulation. **A**, **B** Cell viability was assessed in HepG2 cells transfected with a control vector or an ME1-overexpressing plasmid after 24 h treatment with lenvatinib (**A**) or sorafenib (**B**). **C**–**E** *PTGS2* mRNA (**C**) *CHAC1* mRNA (**D**) and lipid peroxidation byproduct malondialdehyde (MDA) content (**E**) were tested in cells HepG2 cells transfected with a control vector or an ME1-overexpressing plasmid. **F** Cell viability was tested in control or shME1 Huh1 cells following 24 h treatment with lenvatinib (30 µM). **G** Schematic of the induction of lenvatinib-resistant hepatocellular carcinoma cells. Lenvatinib-resistant cells (Huh7-LR) were established from parental Huh7-P cells through a 10-month dose-escalation protocol. The resistant cell line was then continuously maintained in lenvatinib-containing medium for subsequent experiments. **H** Cell viability was tested in Huh7-P and Huh7-LR cells following 24-hour treatment with lenvatinib. **I**, **J** *ME1* mRNA (**I**) and protein (**J**) levels were tested in Huh7-P and Huh7-LR cells. **K** Cell viability was tested in control or shME1 Huh7-LR cells following 24 h treatment lenvatinib. **L** Colony formation assay and quantitative analysis of control or shME1 Huh7-LR cells treated with or without lenvatinib (30 μM). Data were calculated by unpaired two-tailed Student’s *t*-test; *P < 0.05, **P < 0.01, and ***P < 0.001.

Ferroptosis has been closely linked to the targeted therapy for HCC [19]. We found that ME1 overexpression successfully reduced cell death induced by lenvatinib, but not sorafenib (Fig. 5A, B). To further examine whether lenvatinib could directly activate ferroptosis, we measured marker gene expression and MDA contents. The results suggested that ferroptosis induced by lenvatinib was remarkedly inhibited in ME1-overexpressed HepG2 cells (Fig. 5C–E). Moreover, knockdown of ME1 further accelerated lenvatinib-induced cell death, indicating that ME1 regulates the sensitivity of HCC cells to lenvatinib (Fig. 5F and Supplementary Fig. 3F).

Furthermore, we established a lenvatinib-resistant HCC cell model (Huh7-LR) by gradually subjecting the Huh7 cells to increasing concentrations of lenvatinib (Fig. 5G). Compared to parent cells (Huh7-P), Huh7-LR cells exhibited poor responses to lenvatinib as the IC50 value was almost 4.3 times greater (Fig. 5H). In the comparison between the two cell lines, we observed that either mRNA or protein expression of ME1 was remarkably increased in Huh7-LR and Huh7-P cells (Fig. 5I, J). Eventually, ME1 knockdown restored the sensitivity of Huh7-LR cells to lenvatinib by promoting ferroptosis (Fig. 5K, L). Overall, our results prove that ME1 plays an important role in conferring resistance to lenvatinib in HCC.

### NADPH-FSP1-CoQH_2_ pathway drives ferroptotic resistance in ME1-overexpressed HCC

**Fig. 6:**
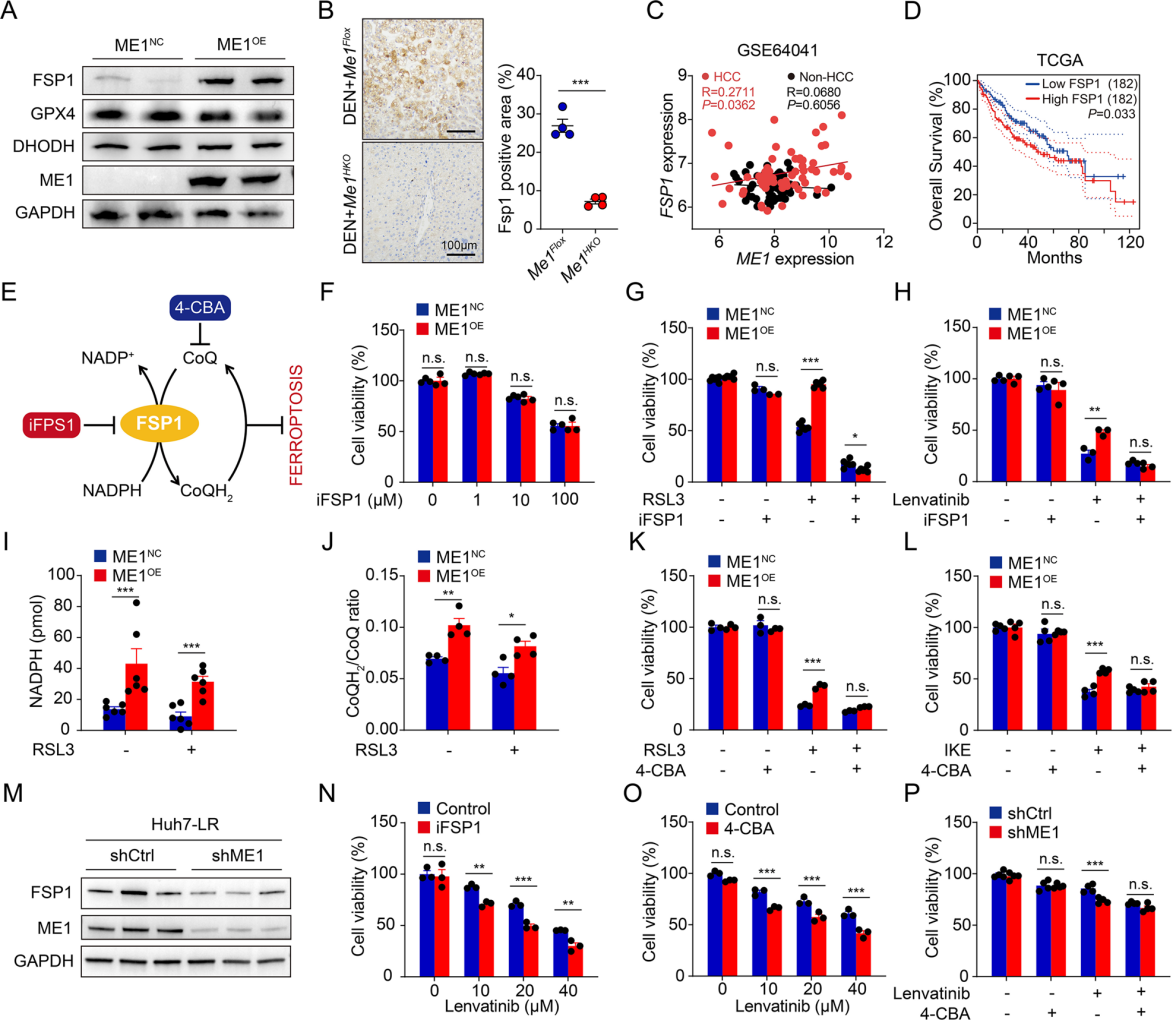
The anti-ferroptotic effect of ME1 is dependent on FSP1. **A** Representative western blotting results of FSP1, GPX4, DHODH, and ME1 in HepG2 cells transfected with a control vector versus an ME1-overexpressing construct. **B** Representative images and quantitative analyses of Fsp1 immunohistochemical staining in livers from DEN-induced *Me1*^*Flox*^ and *Me1*^*HKO*^ mice (*n* = 4 for each group). **C** Correlation of ME1 and FSP1 mRNA level in human HCC and non-HCC samples Biopsies from 60 HCC patients (one tumor biopsy and one non-tumor liver biopsy per patient, *n* = 60 for each group). **D** Overall survival of liver cancer patients (*n* = 182) from the TCGA database was analyzed by FSP1 expression levels. **E** Schematic depicting the ferroptosis regulatory role of the FSP1-CoQ axis. The inhibitors 4-CBA (targeting CoQ) and iFSP1 (targeting FSP1) are indicated. **F** Cell viability was tested in HepG2 cells transfected with control vector or ME1 overexpression vector, treated with different doses of iFSP1 (0–100 μM,) for 24 h. **G**, **H** Cell viability was tested in HepG2 cells transfected with control vector or ME1 overexpression vector, treated with iFSP1(3 μM), combined with RSL3 (20 μM, **G**) or lenvatinib (30 μM, **H**) for 24 h. **I**, **J** Measurement of NADPH (**I**) and CoQH_2_/CoQ ratio (**J**) in ME1^NC^ or ME1^OE^ HepG2 cells treated without or with RSL3 (20 μM) for 24 h. **K**, **L** Cell viability in control or ME1-overexpressing HepG2 cells following 24 h cotreatment with: (**K**) RSL3 (20 μM) + 4-CBA (10 μM), or (**L**) IKE (40 μM) + 4-CBA (10 μM). **M** Representative western blotting results of FSP1 and ME1 in control or shME1 Huh7-LR cells. **N**, **O** Cell viability of Huh7-LR cells treated with lenvatinib, combined with iFSP1 (3 μM, **N**) or 4-CBA (10 μM, **O**) for 24 h. **P** Cell viability was assessed in control or ME1-knockdown (shME1) Huh7-LR cells Data were calculated by unpaired two-tailed Student’s *t*-test; *P < 0.05, **P < 0.01, and ***P < 0.001.

The canonical ferroptotic signaling pathway is mediated by glutathione peroxidase 4 (GPX4)-dependent lipid peroxidation neutralization. RSL3 could covalently bind to the active site of GPX4, and this is the mechanism by which it triggers ferroptosis specifically [17]. However, the resistance of ME1-overexpressed HCC cells to the GPX4 inhibitor led us to consider additional defense pathways. In 2019, FSP1, previously known as AIFM2, was identified as the key component mediating a second ferroptosis suppressive pathway that acts in parallel to GPX4 [20, 21]. More recently, Mao *et al*. linked dihydroorotate dehydrogenase (DHODH) to a mitochondrially localized system inhibiting ferroptosis [22]. Here, we conducted an inspection of these known ferroptosis regulators and found that only FSP1 was significantly upregulated in ME1-overexpressed HCC cells (Fig. 6A). In addition, decreased Fsp1 protein expression was observed in HCC tissues from *Me1*^*HKO*^ mice (Fig. 6B). Interestingly, hepatic *ME1* gene levels correlated positively with the expression of *FSP1* in tumor tissues of HCC patients, and high *FSP1* expression predicted poorer clinical outcome in liver cancer patients (Fig. 6C, D).

ME1-catalyzed biochemical reaction is a major cellular source of NADPH, which supplies the reducing power for various metabolic processes [23, 24]. As an oxidoreductase, FSP1 utilizes NADPH to catalyze the reduction from ubiquinone (CoQ) to ubiquinol (CoQH_2_) on the plasma membrane. CoQH_2_ functions as a radical-trapping antioxidant and thereby inhibits lipid peroxidation and ferroptosis (Fig. 6E). Unlike the obvious effect on other ferroptosis inducers, *ME1* overexpression failed to rescue FSP1 inhibitor (iFSP1)-induced cell death (Fig. 6F and Supplementary Fig. 4A). Furthermore, *ME1* overexpression-induced resistance to RSL3 or lenvatinib was greatly abolished under iFSP1 administration (Fig. 6G, H and Supplementary Fig. 4B, C). Consistently, we found that *ME1* overexpression strongly increased both NADPH levels and CoQH_2_/CoQ ratios in HCC cells (Fig. 6I, J). Then, we tried to artificially limit intracellular CoQ levels by blocking CoQ biosynthesis with 4-chlorobenzoic acid (4-CBA). Similar to iFSP1, 4-CBA treatment largely abolished the ferroptotic resistance conferred by *ME1* overexpression (Fig. 6K, L and Supplementary Figure 4D, E).

In Huh7-LR cells, knockdown of ME1 resulted in a concomitant significant reduction in FSP1 expression (Fig. 6M). Treatment with iFSP1 or 4-CBA partially resensitized these cells to lenvatinib (Fig. 6N, O and Supplementary Fig. 4F). In addition, the reversal of lenvatinib resistance by targeting ME1 is mechanistically dependent on the FSP1-CoQH_2_ axis, as this effect was abrogated by 4-CBA treatment (Fig. 6P). Together, these findings strongly suggest that *ME1* overexpression activates FSP1 through NADPH production and that the CoQ-FSP1 defense system plays a key role in regulating ferroptosis and lenvatinib resistance in HCC cells.

## Discussion

Ferroptosis has been regarded as a targetable vulnerability of cancer, but remains poorly understood in HCC. We demonstrate herein that ME1 exhibits a novel pro-tumorigenic function in HCC by working as a metabolic brake to stop ferroptotic cell death. In normal hepatocytes, ME1 expression helps to protect against tissue injury by maintaining glutathione homeostasis [13]. However, in HCC cells, ME1 expression is significantly upregulated to reprogram NADPH anabolism and thereby promotes FSP1-dependent inhibition of ferroptosis. Loss of *Me1* in mouse livers abolishes ferroptosis suppression, leading to enhanced lipid peroxidation and reduced tumor burden during HCC progression. This metabolic feature also enables HCC cells to survive and progress under lenvatinib treatment, which is the approved first-line therapy for patients with advanced HCC. Targeting ME1 could re-sensitize Huh7-LR cells to lenvatinib, suggesting a promising way to overcome drug resistance and improve treatment response in HCC patients.

ME1 was first associated with carcinogenesis and/or therapy response in 2015 [25, 26]. Since then, *ME1* overexpression has also been demonstrated to promote gastric cancer growth and to predict poor prognosis of breast cancer [10, 12]. Interestingly, spontaneous absence of *ME1* expression was recently found in synovial sarcoma, a rare soft tissue cancer, and could increase the susceptibility to ferroptosis activation [27]. As tumor cells usually require more NADPH supplementation for antioxidation and biosynthesis, the authors indicated that ME1 could suppress ferroptosis through its important function of generating NADPH, which reduces GSSG back to GSH [24, 27]. With GSH as a reducing equivalent, GPX4 is able to neutralize intracellular lipid peroxidation and prevent ferroptotic cell death [28]. However, here we uncovered that ME1 could exhibit the anti-ferroptosis effect in a GSH/GPX4-independent manner. As evidence, exogenous overexpression of *ME1* successfully rescues ferroptosis in HCC cells induced by RSL3, a specific GPX4 inhibitor.

Non-canonical regulatory mechanisms involving in ferroptosis have attracted more and more attention. Although GPX4 is considered the primary factor in controlling ferroptosis, its deletion or inhibition fails to induce ferroptosis in some cancer cells, leading to the hypothesis of alternative resistance pathways. In the context, FSP1 was identified and validated by two independent groups as the second anti-ferroptosis system [20, 21]. In parallel to GPX4, FSP1 works as an NADPH-dependent CoQ reductase to regenerate the reduced CoQ form (CoQH_2_ or ubiquinol), which is a lipid-soluble antioxidant and effectively prevents lipid peroxidation in the plasma membrane.

Although iFSP1 treatment alone is usually not sufficient to trigger ferroptosis in multiple cancer cell lines, it should be noted that FSP1 may have greater potential to become an anti-cancer target than GPX4 in clinical application [29]. GPX4 is an essential gene in development and physiological function, as its deletion leads to early embryonic lethality or a rapid fatal phenotype (in the context of inducible gene knockout) [30, 31]. While *Fsp1*-deficient mice are fully viable [32]. Thus, FSP1 inhibition probably results in less injury in normal tissues compared to GPX4 inhibition. In the present study, we demonstrated that FSP1 is highly expressed in *ME1*-overexpressing HCC cells and that FSP1 is also required for drug resistance, further supporting its role as a promising target for HCC therapy.

Targeted therapy has been a preferred option for HCC patients who are unsuitable for surgical resection. Sorafenib, a multi-kinase inhibitor targeting Raf and several other oncogenic kinases, is the first approved agent for advanced HCC patients [33]. After the discovery of ferroptosis, it was quickly proposed to work as a ferroptosis inducer in the next year [34]. Subsequently, numerous studies repeatedly reported the phenomenon and hypothesized multiple possible mechanisms involved [35, 36]. However, Zheng *et al*. demonstrated that sorafenib could not trigger ferroptotic cell death in a series of cancer cell lines [37]. In this study, *ME1* overexpression failed to rescue sorafenib-induced cell death, indirectly supporting the above conclusion. Previously, a study drew an in vitro link between cytotoxicity induced by lenvatinib, the newer first-line drug for unresectable HCC, and ferroptosis [38]. Here, we found that overexpression of *ME1* suppressed lipid peroxidation, which correlated with lenvatinib-induced cell death. Whit this pro-ferroptotic feature, lenvatinib, rather than sorafenib, may be a more potent treatment option in the future.

Although lenvatinib has become a standard treatment at present, its efficacy and therapeutic duration in the clinic are severely hampered by drug resistance. The precise mechanisms involved in lenvatinib resistance are complex and still remain largely unknown thus far. Recently, there are a few reports highlighting the notable role of metabolic reprogramming in the development of lenvatinib resistance. For instance, Wang et al. reported that high expression of acylphosphatase 1 (ACYP1) may contribute to lenvatinib resistance depending on the Warburg effect, a phenomenon in which cancer cells rely primarily on aerobic glycolysis to produce the energy needed for proliferation [39]. Similarly, enhanced cholesterol biosynthesis, which is mediated by transcription factor SREBP2, helps HCC cells to achieve lenvatinib resistance [40]. In addition, there are no reliable biomarkers to predict the treatment response to lenvatinib. In the present study, we revealed for the first time that ME1 expression was significantly upregulated in the lenvatinib-resistant HCC cells. Inhibition of ME1 successfully re-sensitized these cells to lenvatinib-induced growth inhibition and cell death. These results indicate that ME1 might potentially serve as a biomarker for predicting lenvatinib response and pave the way for personalized HCC therapy in the future.

In summary, the present study demonstrates that ME1 is highly expressed in HCC, especially in lenvatinib-resistant cancer cells, and is associated with poor prognosis. Loss of ME1 remarkably suppresses hepatocellular carcinogenesis and drug resistance through FSP1-dependent regulation of ferroptosis. These findings highlight the biological significance of hepatic ME1 in HCC development as well as its role as a therapeutic target to reverse lenvatinib resistance.

## Supplementary information

SUPPLEMENTAL MATERIAL

Histology images

WE gels

## Data Availability

The raw data that support the findings of this study are available from the corresponding author upon reasonable request.
