# Supplementary material for: Malic enzyme 1 contributes to tumorigenesis and lenvatinib resistance in hepatocellular carcinoma via FSP1-dependent ferroptosis evasion
Source: Cell Death Dis. 2026 Mar 25;17(1):360. doi: 10.1038/s41419-026-08572-w (PMC13039963; doi:10.1038/s41419-026-08572-w)
Supplement: Supplementary file 1 — SUPPLEMENTAL MATERIAL [file 41419_2026_8572_MOESM1_ESM.docx]

**Malic Enzyme 1 Contributes to Tumorigenesis and Lenvatinib Resistance in Hepatocellular Carcinoma via FSP1-dependent Ferroptosis Evasion**

Wu *et al.*

This file includes:

Supplementary Tables 1-3

Supplementary Figures 1-4

**Supplementary Table 1. Chemicals used in this study.**

| **Reagents** | **Source** | **Catalog #** |
| --- | --- | --- |
| RSL3 | MedChemExpress（MCE） | HY-100218A |
| IKE | MedChemExpress（MCE） | HY-114481 |
| FINO2 | MedChemExpress（MCE） | HY-129457 |
| Auranofin | MedChemExpress（MCE） | HY-B1123 |
| FeSO_4_ | Sigma-Aldrich | 7782-63-0 |
| Diethylnitrosamine（DEN） | Sigma-Aldrich | N0258 |
| Lenvatinib | Selleck Chemicals | S1164 |
| Sorafenib | Selleck Chemicals | S7397 |
| iFSP1 | MedChemExpress（MCE） | HY-136057 |
| 4-chlorobenzoic acid (4-CBA) | Sigma-Aldrich | 135585 |

**Supplementary Table 2. The shRNA and qPCR primer sequences used in this study.**

| **qPCR primers** | |
| --- | --- |
| *GAPDH* Forward | GGAGCGAGATCCCTCCAAAAT |
| *GAPDH* Reverse | GGCTGTTGTCATACTTCTCATGG |
| *ME1* Forward | CTGCTGACACGGAACCCTC |
| *ME1* Reverse | GATCTCCTGACTGTTGAAGGAAG |
| *PTGS2* Forward | TAAGTGCGATTGTACCCGGAC |
| *PTGS2* Reverse | TTTGTAGCCATAGTCAGCATTGT |
| *CHAC1* Forward | GAACCCTGGTTACCTGGGC |
| *CHAC1* Reverse | CGCAGCAAGTATTCAAGGTTGT |
| **shRNA sequences** | |
| shME1 | CCGG-GCCTTCAATGAACGGCCTATT-CTCGAG-AATAGGCCGTTCATTGAAGGC-TTTTTT |
| shCtrl | CCGG-GATTCTCCGAACGTGTCACGT-CTCGAG-ACGTGACACGTTCGGAGAATC-TTTTTT |

**Supplementary Table 3. Antibodies used in this study.**

| **Antigen** | **Source** | **Catalog #** | **RRID** | **Application** |
| --- | --- | --- | --- | --- |
| ME1 | Abcam | AB97445 | AB_10679994 | WB, IHC |
| 4HNE | Abcam | AB46545 | AB_722490 | IHC |
| FSP1 | ABclonal | A22278 | AB_2878756 | WB, IHC |
| DHODH | Proteintech | 14877-1-AP | AB_2091723 | WB |
| GAPDH | Proteintech | 60004-1-Ig | AB_2107436 | WB |
| GPX4 | Proteintech | 67763-1-Ig | AB_2909469 | WB |
| Mouse IgG (Immunoglobulin G) molecule | Proteintech | SA00001-1 | AB_2722565 | WB |
| Rabbit IgG (Immunoglobulin G) molecule | Proteintech | SA00001-2 | AB_2722564 | WB |


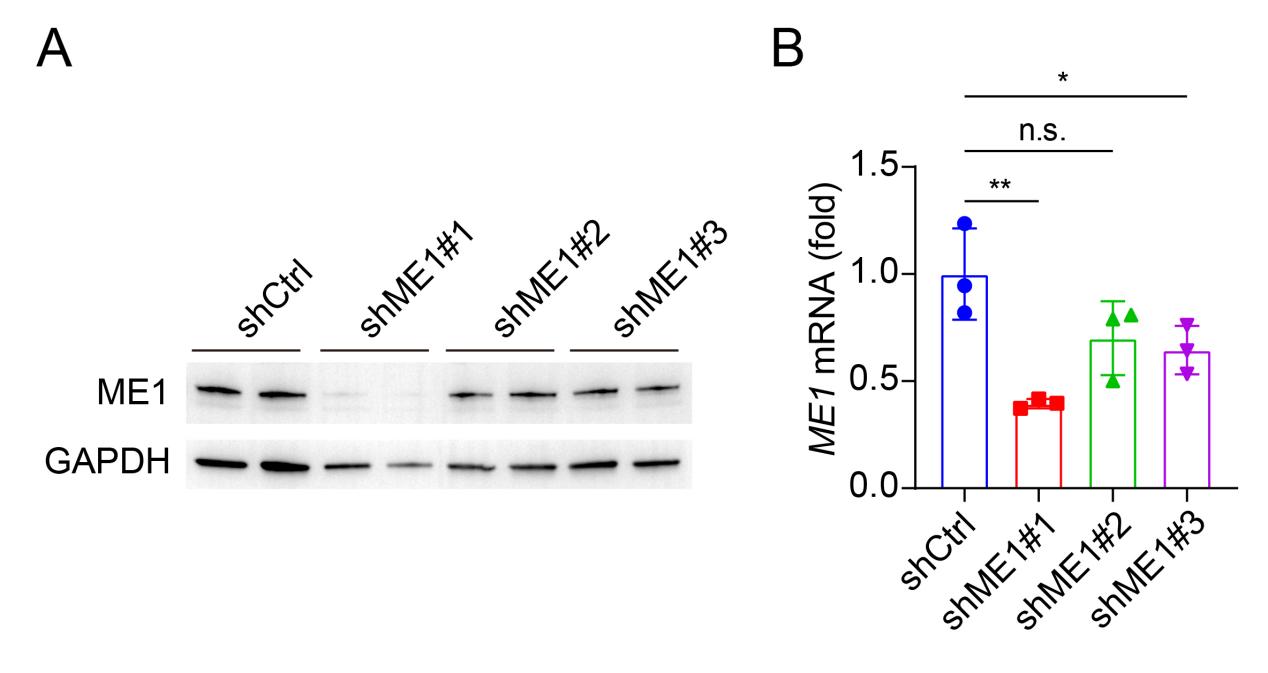


**Supplementary Figure 1. Validation of ME1 knockdown efficiency in Huh7 cells.**

Huh1 cells were transfected with control shRNA (shCtrl) or one of three distinct shRNAs targeting ME1 (shME1#1, shME1#2, shME1#3). (A) Western blot analysis of ME1 protein levels, with GAPDH used as a loading control. (B) qPCR assessment of *ME1* mRNA expression, normalized to GAPDH. Data are presented as mean ± SEM; **P* < 0.05, ***P* < 0.01 versus shCtrl by two-tailed Student’s *t*-test. Among the three shRNAs, shME1#1 exhibited the most potent knockdown efficiency and was selected for subsequent functional experiments.


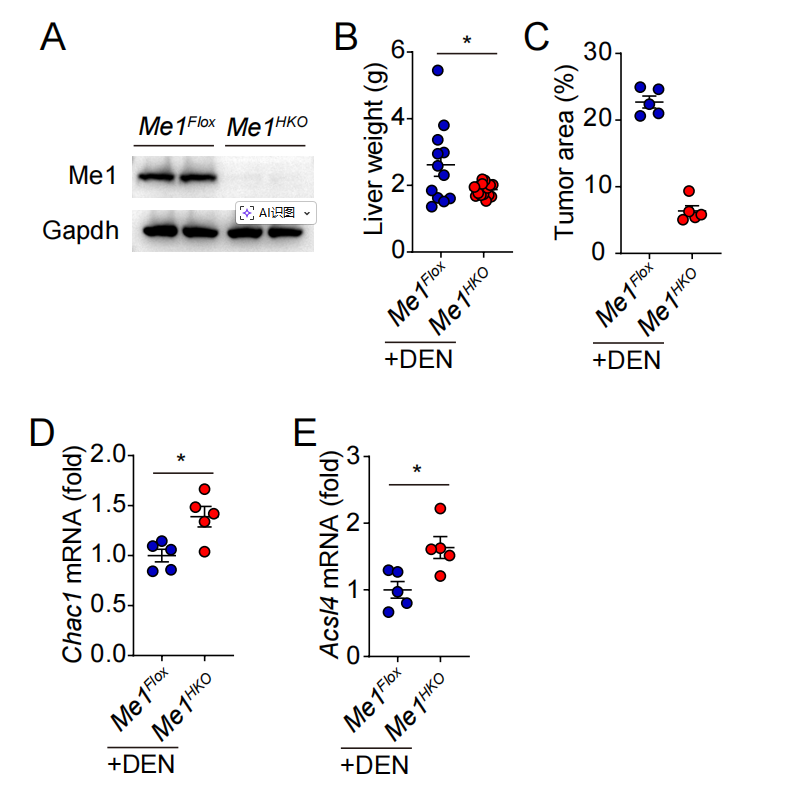


**Supplementary Figure 2. Hepatocyte-specific deletion of Me1 attenuates DEN-induced HCC and ferroptosis in mice.**

1. Western blot analysis confirming efficient knockout of Me1 protein in livers of *Me1^HKO^* mice compared to *Me1^Flox^* controls, with Gapdh as a loading control.
2. Liver weight of *Me1^HKO^* and *Me1^Flox^* mice 8 months after DEN administration.
3. Quantification of tumor area fraction in H&E-stained liver sections from DEN-treated *Me1^HKO^* and *Me1^Flox^* mice.

(D, E) qPCR analysis of hepatic mRNA levels of ferroptosis markers Chac1 (D) and Acsl4 (E) in DEN-treated *Me1^HKO^* and *Me1^Flox^* mice, normalized to Gapdh.

Data are presented as mean ± SEM; **P* < 0.05, ***P* < 0.01 by two-tailed Student’s *t*-test.


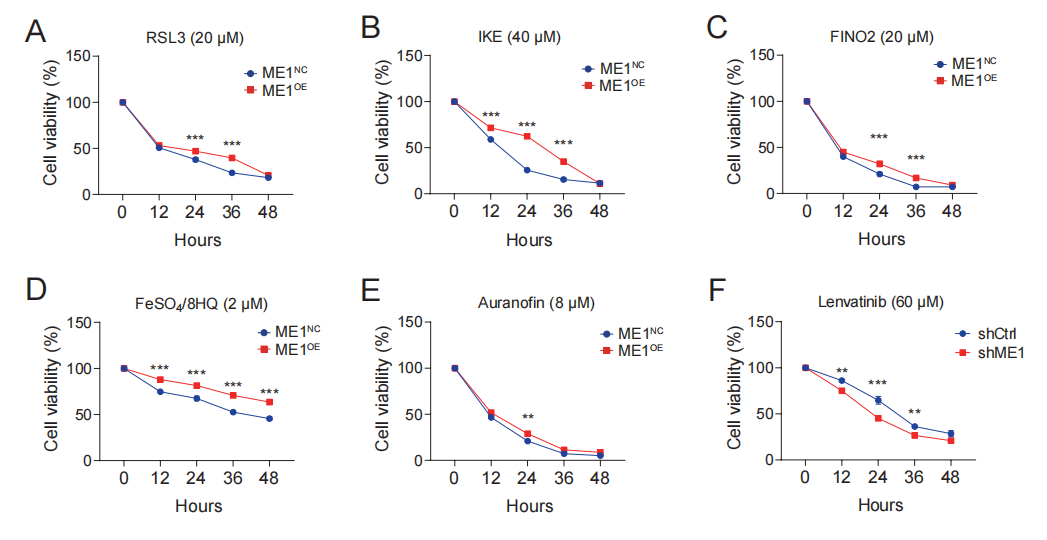


**Supplementary Figure 3. ME1 protects HCC cells against ferroptosis.**

(A)Time-lapse cell viability analysis of ME1-overexpressing HepG2 cells treated with 20 μM RSL3 over 48 hours.

(B)Time-lapse cell viability analysis of ME1-overexpressing HepG2 cells treated with 40 μM IKE over 48 hours.

(C)Time-lapse cell viability analysis of ME1-overexpressing HepG2 cells treated with 20 μM FINO2 over 48 hours.

(D)Time-lapse cell viability analysis of ME1-overexpressing HepG2 cells treated with 2 μM FeSO_4_/8HQ over 48 hours.

(E)Time-lapse cell viability analysis of ME1-overexpressing HepG2 cells treated with 8 μM auranofin over 48 hours.

(F)Time-lapse cell viability analysis of control or shME1 Huh1 cells treated with 60 μM lenvatinib over 48 hours.


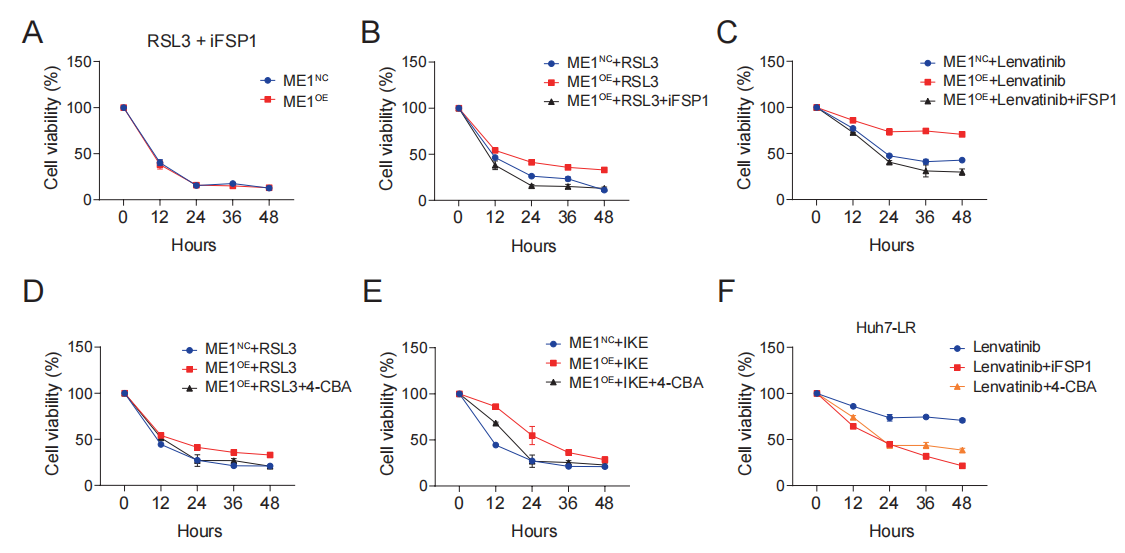


**Supplementary Figure 4. The anti-ferroptotic effect of ME1 is dependent on FSP1/CoQH_2_ axis.**

(A)Time-lapse cell viability analysis of control or ME1-overexpressing HepG2 cells treated with RSL3 plus iFSP1 over 48 hours.

(B)Time-lapse cell viability analysis of ME1-overexpressing HepG2 cells treated with RSL3, combined with iFSP1 over 48 hours.

(C)Time-lapse cell viability analysis of ME1-overexpressing HepG2 cells treated with lenvatinib, combined with iFSP1 over 48 hours.

(D,E)Time-lapse cell viability analysis of ME1-overexpressing HepG2 cells treated with RSL3 (D) or IKE (E), combined with 4-CBA over 48 hours.

1. Time-lapse cell viability analysis of Huh7-LR cells treated with lenvatinib, combined with iFSP1 or 4-CBA over 48 hours.
