# Supplementary material for: Malic enzyme 1 contributes to tumorigenesis and lenvatinib resistance in hepatocellular carcinoma via FSP1-dependent ferroptosis evasion
Source: Cell Death Dis. 2026 Mar 25;17(1):360. doi: 10.1038/s41419-026-08572-w (PMC13039963; doi:10.1038/s41419-026-08572-w)

# Figure 2I

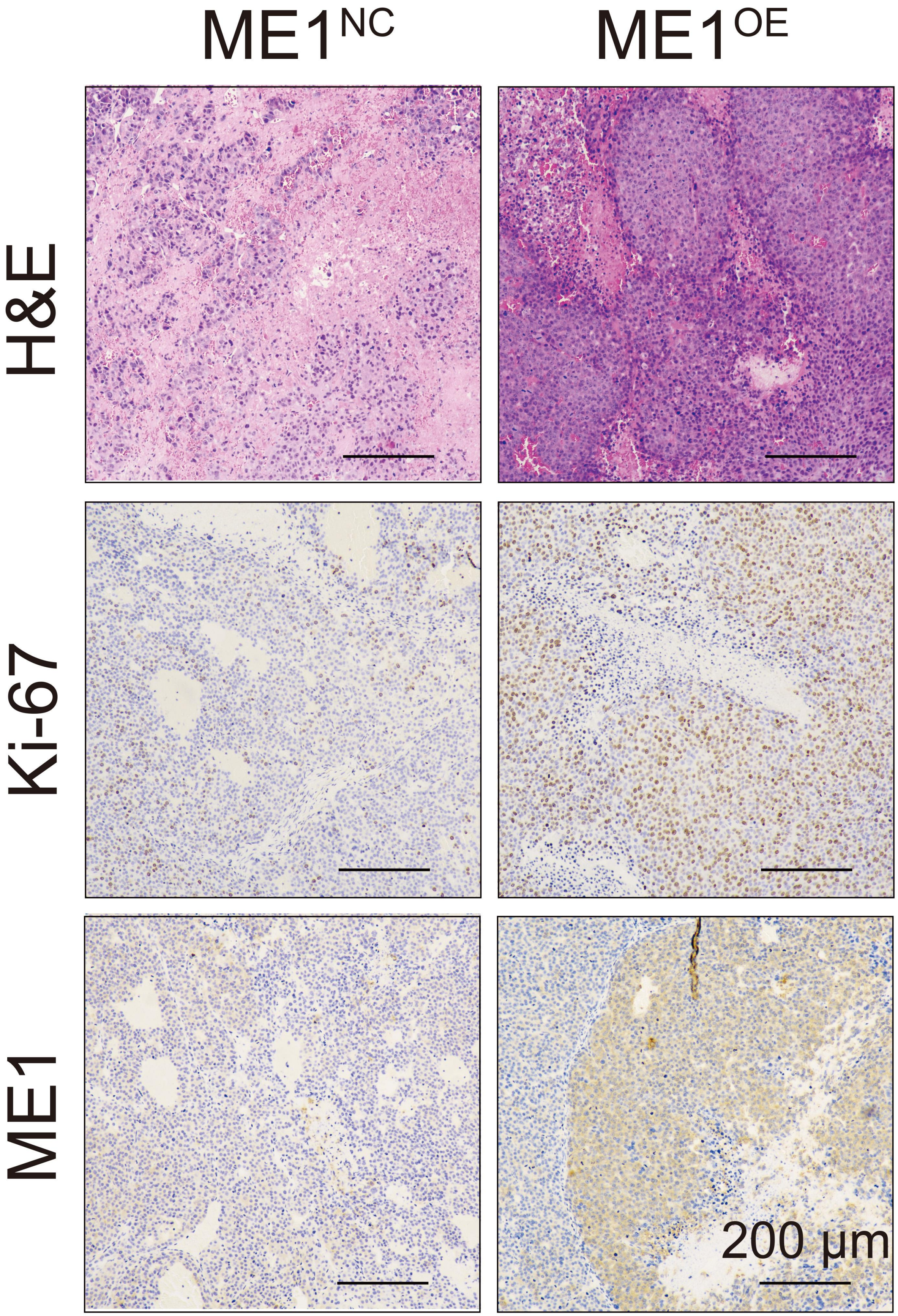

# Figure 3K

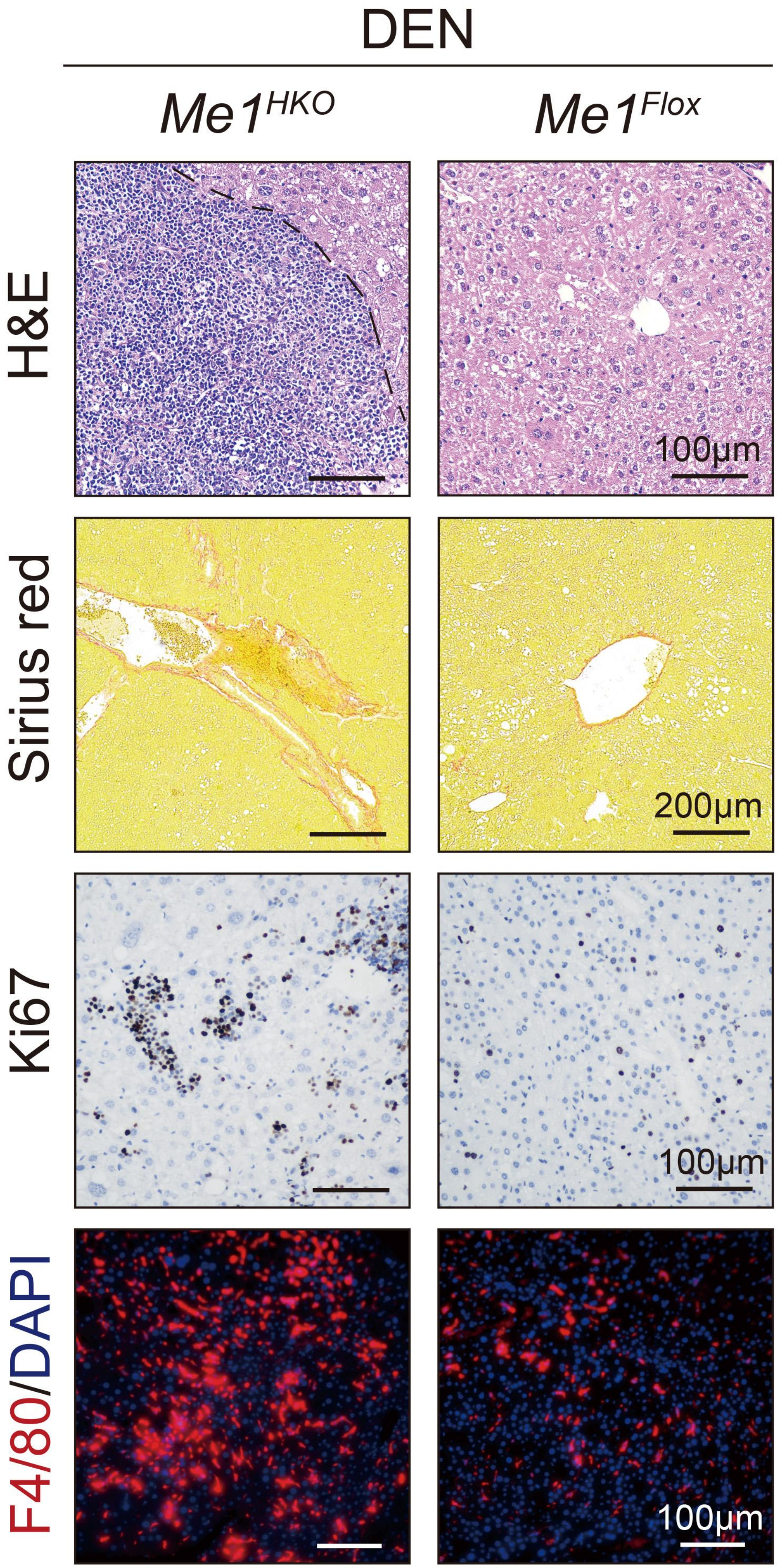

DEN
$Me1^{HKO}$
$Me1^{Flox}$
H&E
Sirius red
Ki67
F4/80/DAPI
100μm
200μm
100μm
100μm

# Figure 4J

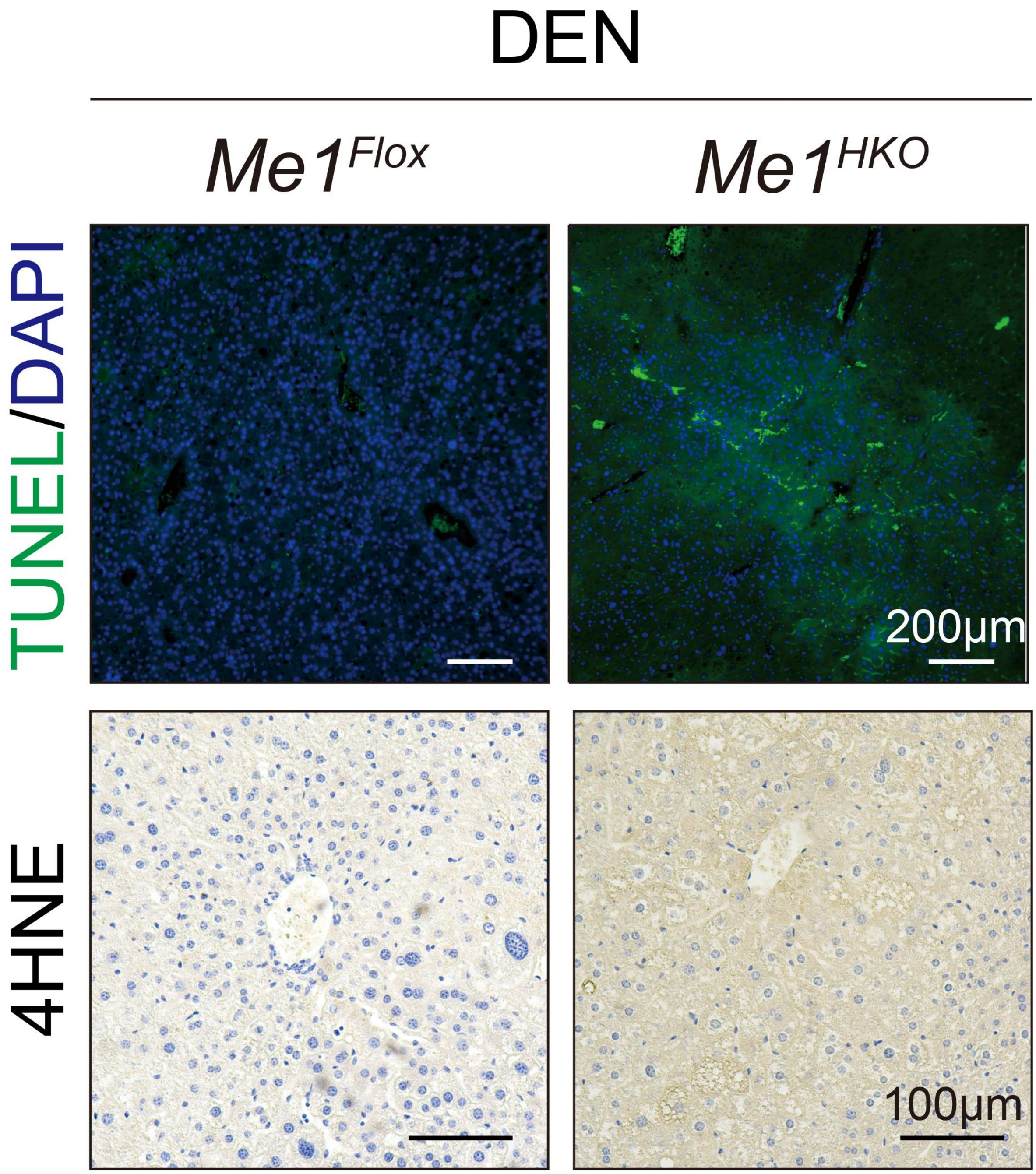

# Figure 6B

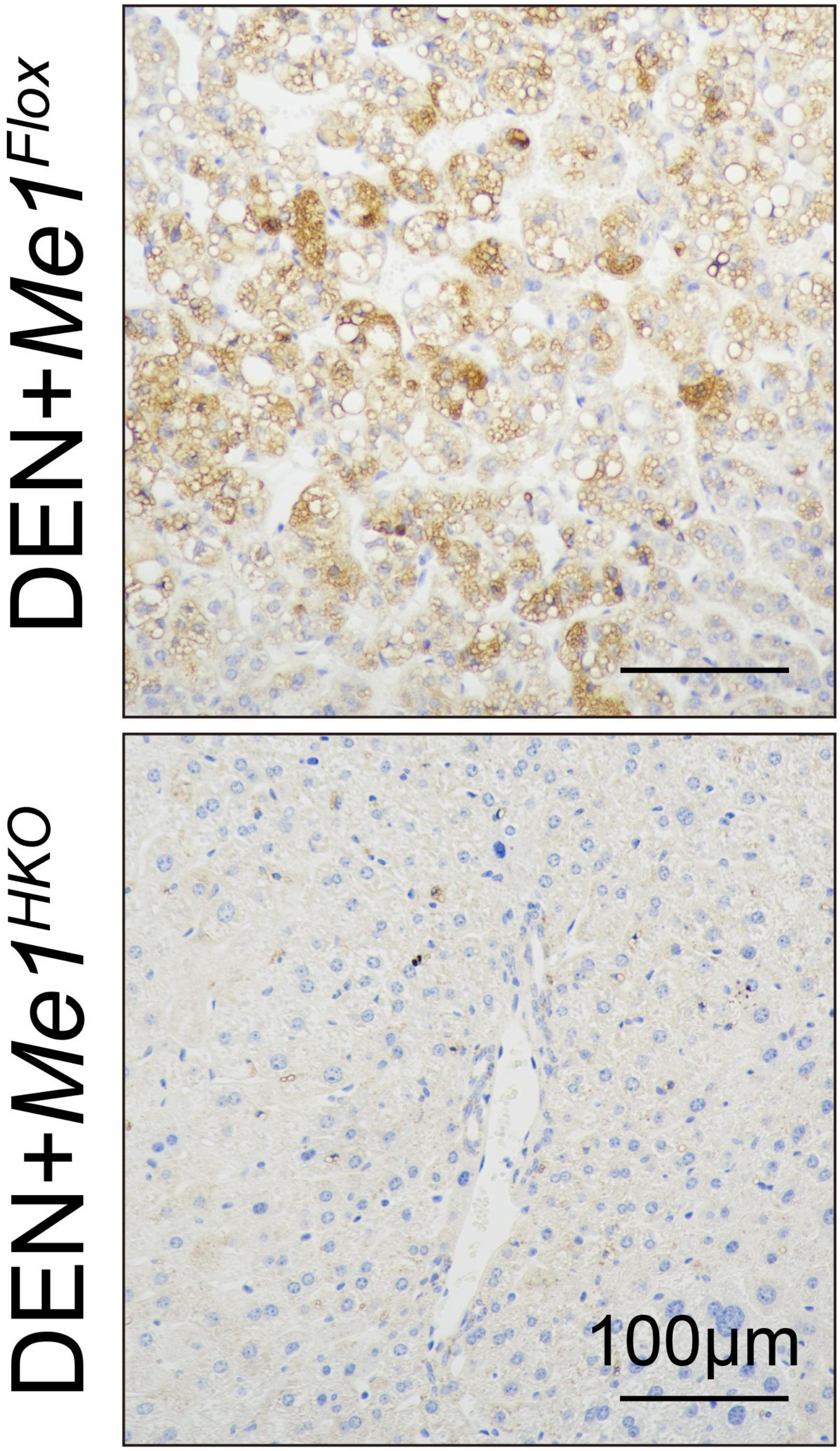

DEN+*Me1*$^{Flox}$

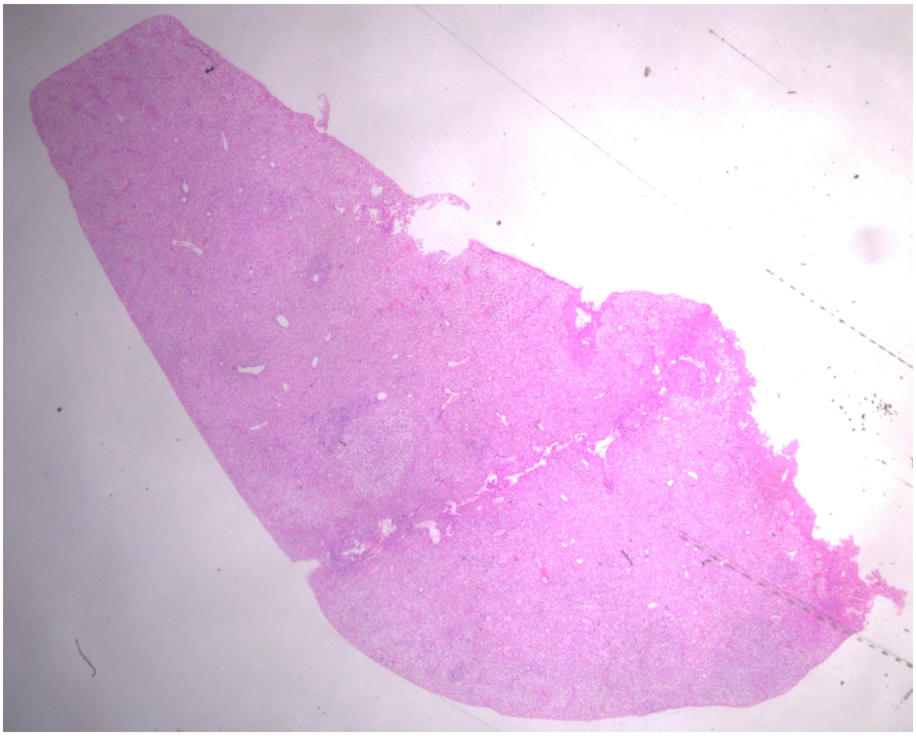

DEN+*Me1*$^{HKO}$

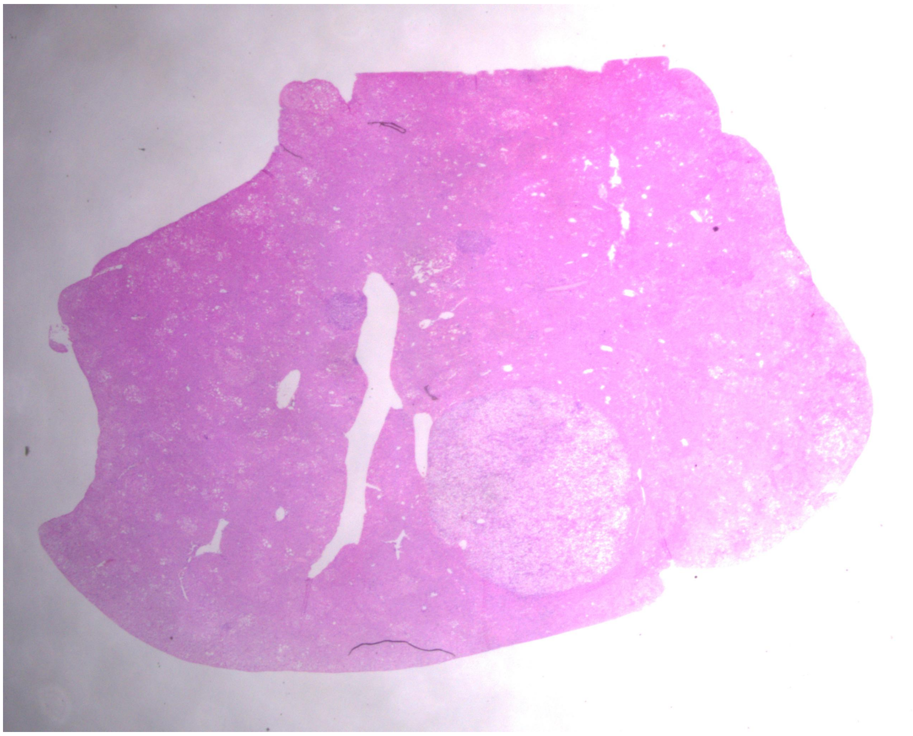

Supplement: Supplementary file 2 — Histology images [file 41419_2026_8572_MOESM2_ESM.pdf]
