# Supplementary material for: Malic enzyme 1 contributes to tumorigenesis and lenvatinib resistance in hepatocellular carcinoma via FSP1-dependent ferroptosis evasion
Source: Cell Death Dis. 2026 Mar 25;17(1):360. doi: 10.1038/s41419-026-08572-w (PMC13039963; doi:10.1038/s41419-026-08572-w)

Figure 2C

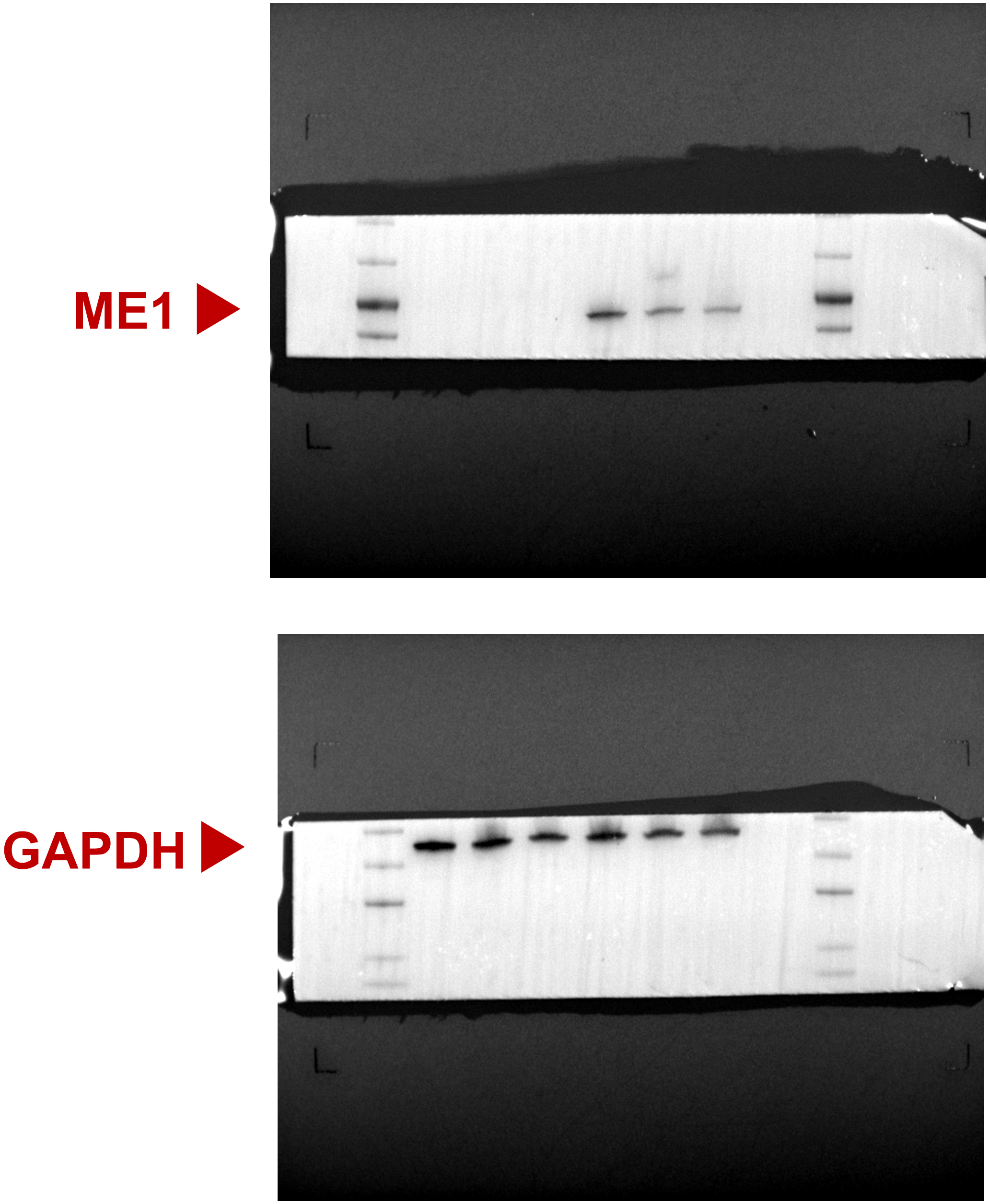

# Figure 2L

**ME1** ▶

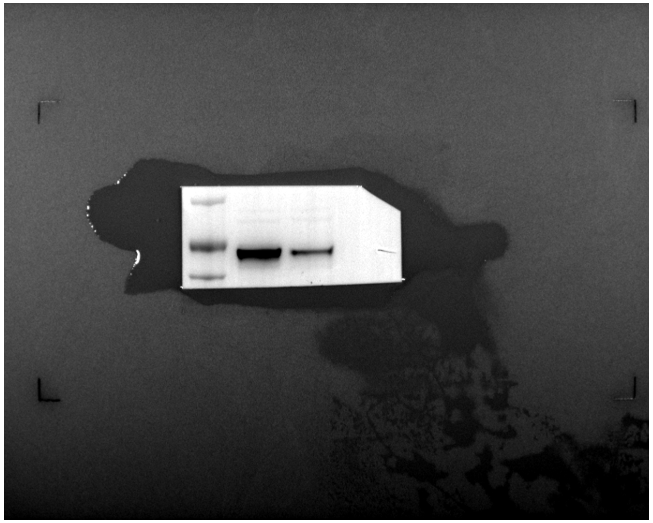

**GAPDH** ▶

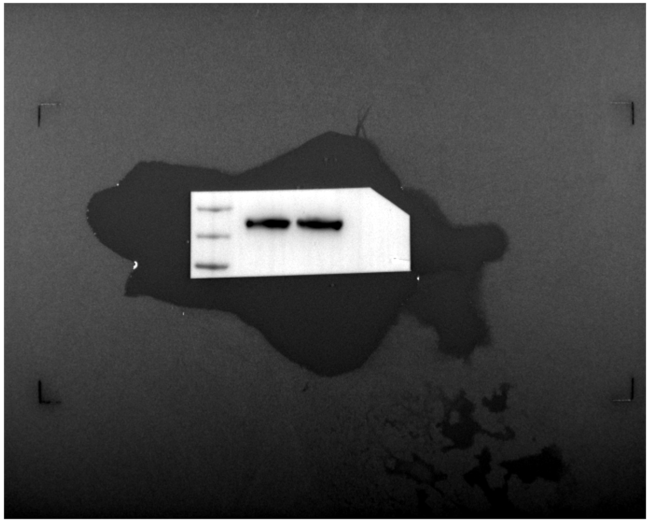

Figure 5J

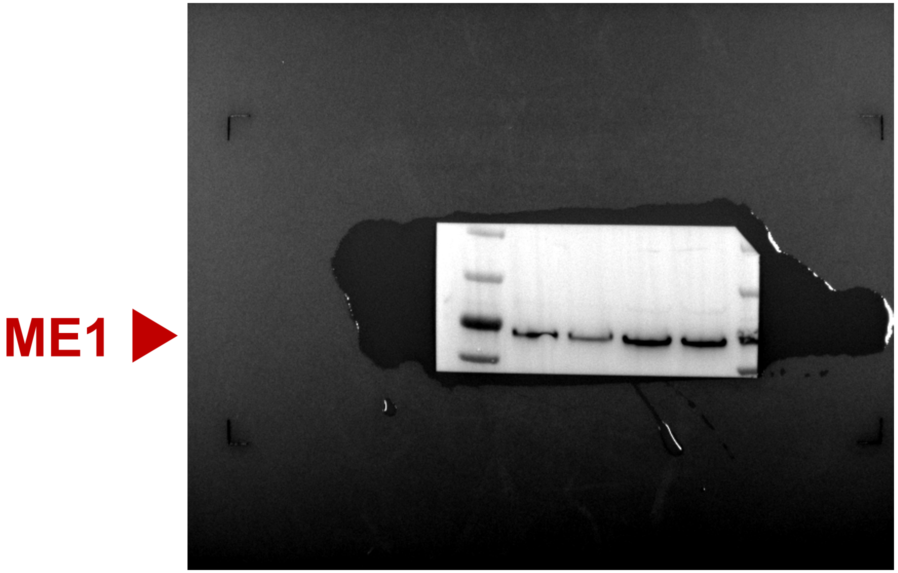


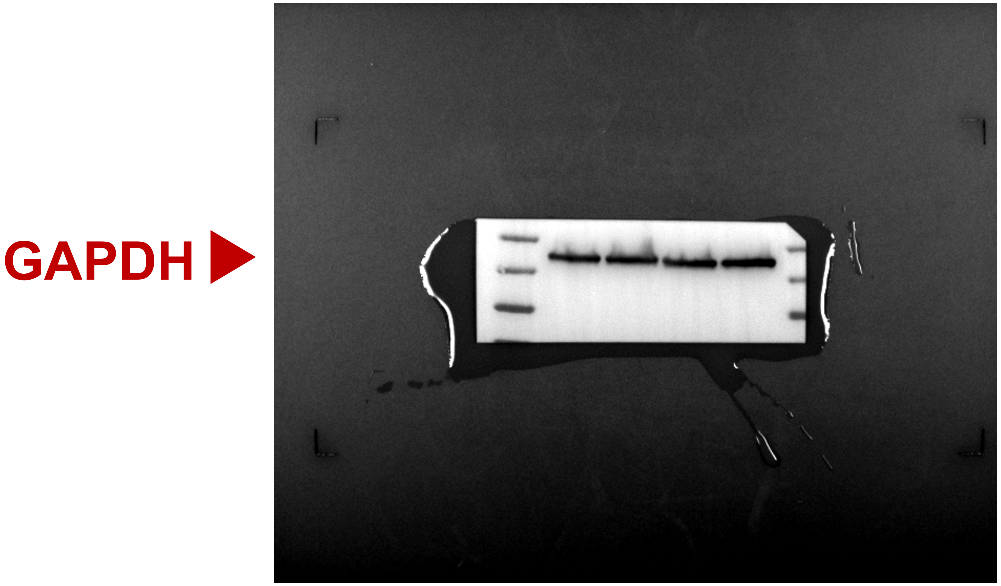

Figure 6A

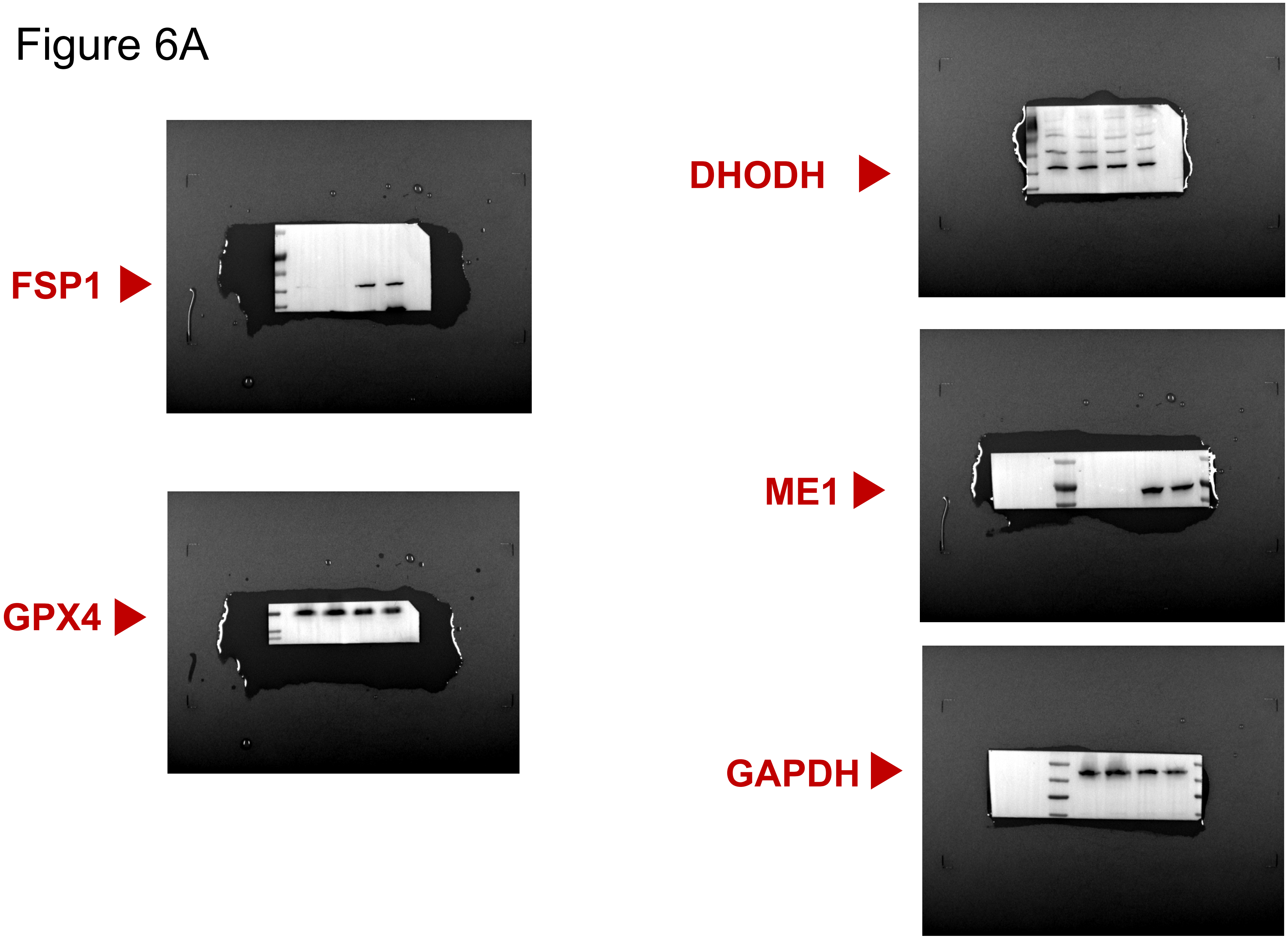

# Figure 6M

FSP1

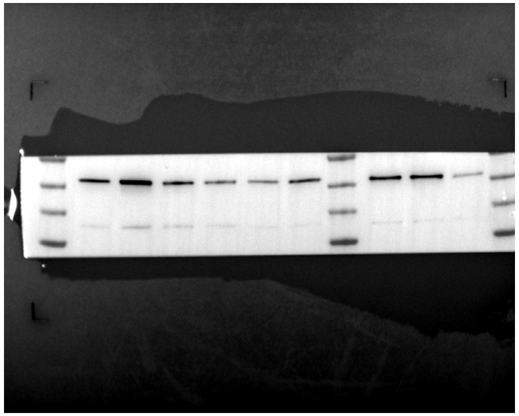

ME1

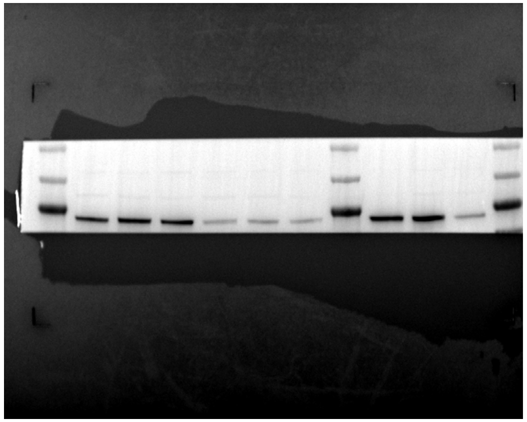

GAPDH

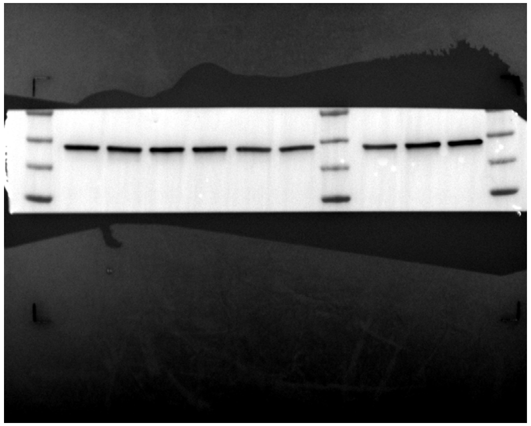

Supplement: Supplementary file 3 — WE gels [file 41419_2026_8572_MOESM3_ESM.pdf]
